# The mitochondrially targeted antioxidant MitoQ protects the intestinal barrier by ameliorating mitochondrial DNA damage via the Nrf2/ARE signaling pathway

**DOI:** 10.1038/s41419-018-0436-x

**Published:** 2018-03-14

**Authors:** Qiongyuan Hu, Jianan Ren, Guanwei Li, Jie Wu, Xiuwen Wu, Gefei Wang, Guosheng Gu, Huajian Ren, Zhiwu Hong, Jieshou Li

**Affiliations:** 1 Department of Surgery, Jinling Hospital, Medical School of Nanjing University, Nanjing, China; 2 Lab for Trauma and Surgical Infection, Nanjing, China; 3 Medical school of Nanjing Medical University, Nanjing, China

## Abstract

Disruption of the mucosal barrier following intestinal ischemia reperfusion (I/R) is life threatening in clinical practice. Mitochondrial dysfunction and oxidative stress significantly contribute to the early phase of I/R injury and amplify the inflammatory response. MitoQ is a mitochondrially targeted antioxidant that exerts protective effects following I/R injury. In the present study, we aimed to determine whether and how MitoQ protects intestinal epithelial cells (IECs) from I/R injury. In both in vivo and in vitro studies, we found that MitoQ pretreatment downregulated I/R-induced oxidative stress and stabilized the intestinal barrier, as evidenced by MitoQ-treated I/R mice exhibiting attenuated intestinal hyperpermeability, inflammatory response, epithelial apoptosis, and tight junction damage compared to controls. Mechanistically, I/R elevated mitochondrial 8-hydroxyguanine content, reduced mitochondrial DNA (mtDNA) copy number and mRNA transcription levels, and induced mitochondrial disruption in IECs. However, MitoQ pretreatment dramatically inhibited these deleterious effects. mtDNA depletion alone was sufficient to induce apoptosis and mitochondrial dysfunction of IECs. Mitochondrial transcription factor A (TFAM), a key activator of mitochondrial transcription, was significantly reduced during I/R injury, a phenomenon that was prevented by MitoQ treatment. Furthermore, we observed that thee protective properties of MitoQ were affected by upregulation of cellular antioxidant genes, including HO-1, NQO-1, and γ-GCLC. Transfection with Nrf2 siRNA in IECs exposed to hypoxia/reperfusion conditions partially blocked the effects of MitoQ on mtDNA damage and mitochondrial oxidative stress. In conclusion, our data suggest that MitoQ exerts protective effect on I/R-induced intestinal barrier dysfunction.

## Introduction

Intestinal ischemia reperfusion (I/R) is one of the most common, critical events due to acute mesenteric ischemia, hemorrhagic shock, trauma, sepsis, burns, and surgical procedures^1^. Intestinal I/R increases intestinal permeability and demolishes mucosal barrier function^2^. Damaging or overwhelming the intestinal barrier provides route for viable bacteria and their antigens from the luminal environment to enter the circulation and mesenteric lymph, leading to systemic response syndrome (SIRS) or multiple organ dysfunction syndrome (MODS)^3^. Maintenance or repair of the intestinal mucosal barrier is, therefore, a key target for I/R-associated rescue in critically ill patients.

Oxidative stress plays a critical role during the pathogenesis of intestinal I/R. Previous research has suggested that the imbalance between oxygen delivery and oxygen demand induces production of reactive oxygen species (ROS), with mitochondria hypothesized to be the major source for these ROS^4^. Excessive ROS triggers activation of various signaling pathway, induces apoptosis or necrosis, increases inflammatory responses, and impairs gut barrier function during intestinal I/R injury^3,5,6^.

Mitochondrial DNA (mtDNA) is double-stranded circular 16.5 kbp DNA containing 37 genes, all of which are involved in oxidative phosphorylation and normal mitochondrial function^7^. mtDNA is vulnerable to accumulating oxidative stress because of its proximity to mitochondrial ROS and the lack of protective histones^8^. Once mtDNA is damaged, critical proteins encoded by respiratory chain genes becomes deficient, increasing ROS formation and mitochondrial disruption^9,10^. Accumulating evidence indicates that increased ROS production and subsequent mtDNA damage are critical for the pathogenesis of I/R injury^10,11^. However, it is unknown whether or how oxidative injury to mtDNA participates in intestinal I/R and mucosal barrier disruption.

**Fig. 1:**
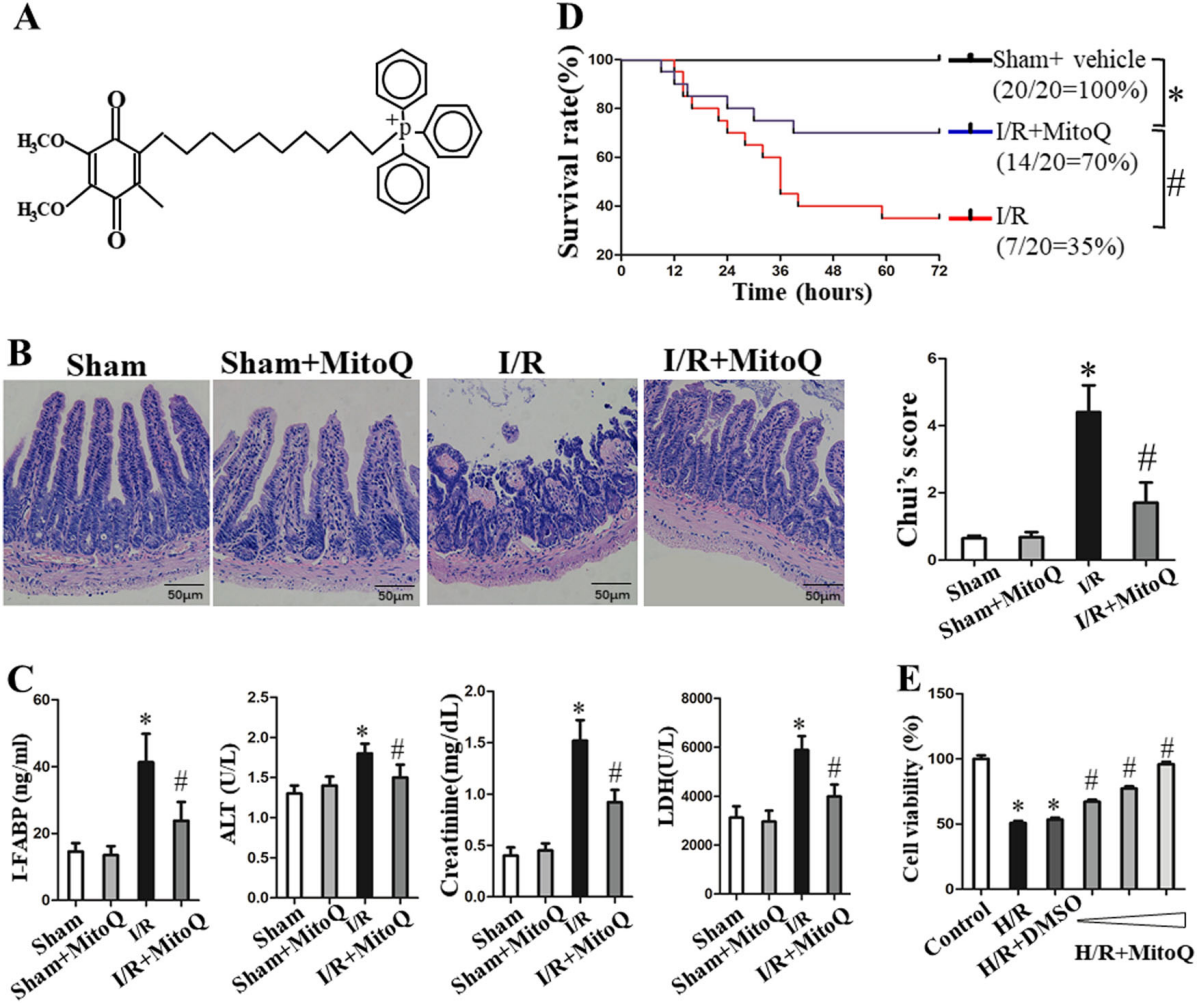
MitoQ protects against intestinal I/R injury in mice. **a** Chemical structure of MitoQ. **b** Representative images of intestinal histology (H&E staining, original magnification ×200) and Chiu’s score of the intestine following intestinal I/R. **c** Levels of serum I-FABP, ALT, creatinine, and LDH. **d** The Kaplan–Meier survival curves compared by the log-rank test. **e** Cell viability in H/R-treated IEC-6 cells analyzed with CCK-8 assay. Data are expressed as the mean ± SD. ^*^P < 0.05 vs sham or control group; ^#^*P* < 0.05 vs I/R or H/R group

The mitochondrially targeted antioxidant MitoQ (shown in Fig. 1a) comprises a lipophilic triphenylphosphonium cation and coenzyme Q10, making it several hundred-fold more potent than untargeted antioxidants in blocking ROS and preventing mitochondrial oxidative damage^12^. MitoQ has been extensively therapeutically utilized in various diseases including neurodegenerative disease, cardiac hypertrophy, and liver fibrosis^12^. Recent studies suggest that MitoQ also protects mtDNA from oxidative damage in response to I/R injury^13,14^. Therefore, we hypothesized that MtioQ could protect the intestinal barrier following I/R by ameliorating mtDNA damage.

Nuclear factor E2-related factor 2 (Nrf2), a critical transcription factor involved in cellular antioxidant and anti-inflammatory responses, is widely expressed in all human tissues^15^. Under normal conditions, Nrf2 maintained at low levels in the cytoplasm by Keap-1-dependent proteasome degradation. Activated Nrf2 translocates to the nucleus, combines with the antioxidant response element (ARE), and regulates expression of cytoprotective genes^16^. The Nrf2/ARE pathway is widely regarded as a multi-organ protector owing to its anti-oxidative and cytoprotective functions and has been shown to protect against gut inflammation and epithelial barrier dysfunction^15,17–19^. Notably, a recent study revealed that MitoQ exerts beneficial effects in tubular injury via mitophagy and that mitochondrial dysfunction was mediated by Nrf2 signaling^20^. Furthermore, the Nrf2–ARE activator carnosic acid was suggested to decrease mitochondrial dysfunction following traumatic brain injury in mice^21^. Here, we hypothesize that MitoQ may protect against intestinal I/R injury by ameliorating mitochondrial dysfunction and mtDNA damage through activation of Nrf2 signaling.

In the present study, we investigated the role of MitoQ following intestinal I/R injury, and our results suggest that MitoQ enhances intestinal barrier integrity by activating the Nrf2/ARE pathway and preventing damage to mtDNA from oxidative stress, which was associated with stabilization of mitochondrial transcription factor A (TFAM) and a decrease in mitochondrial ROS production. Given that mtDNA damage detrimentally contributes to induction of high ROS and mitochondrial dysfunction, MitoQ pretreatment could be a potent strategy to prevent this early stage of I/R injury.

## Methods

### Animals

Male C57BL/6 mice (aged 8 weeks) obtained from the Model Animals Research Center of Nanjing University were maintained under specific conditions in a temperature controlled room. The animal study was designed and performed in accordance with the principles of the Declaration of Helsinki and with approval from the institutional animal ethical committee of Jinling Hospital.

### Ischemia/reperfusion model

Anesthetic management and experimental intestinal I/R establishment were performed as previously described. In brief, mice were anesthetized intraperitoneally with chloral hydrate. After midline laparotomy, the superior mesenteric artery of I/R mice was identified and occluded with an atraumatic microvascular clamp for 30 mins followed by 6 h of reperfusion. Mice were randomly allocated into the following groups: (1) sham operation; (2) sham operation+MitoQ; (3) intestinal I/R+vehicle; and (4) I/R+MitoQ. MitoQ (4 mg/kg; added as MitoQ adsorbed to β-cyclodextran) (100 μl 0.9% saline) was injected into the tail vein 15 min before the onset of ischemia.

The hypoxia/reoxygenation (H/R) model was adopted using IEC-6 cells, which mimic I/R injury in vivo. In brief, IEC-6 cells were starved in serum-free medium and maintained at 37 °C in a humidified atmosphere. To establish a hypoxic condition, a microaerophilic system (5% CO_2,_1% O_2_, and 94%N_2_) was used to incubate IEC-6 cells for 12 h. Cells were then transferred to normal conditions to achieve reoxygenation. IEC-6 cells were treated with 0.1 μM, 0.5 μM, or 1.0 μM MitoQ for 6 h prior to H/R treatment. DMSO was used as a control.

### Histopathological assessment of intestines

After reperfusion, 1 cm of small intestine was fixed in 10% formalin and embedded in paraffin, and 4 µm sections were stained with hematoxylin and eosin (HE) for light microscopy. The histological score of intestine injury was assessed base on the method by Chiu et al^22^ with the following modifications: 0, no injury; 1, sub-epithelial space of villous tip; 2, loss of mucosal lining in the villous tip; 3, loss of less than half the villous structure; 4, loss of more than half of the villous structure; and 5, transmural necrosis. Sections blindly were evaluated.

### Cell viability

Cell viability was measured using a commercially available Cell Counting Kit (CCK-8) (P.R.C, KeyGEN Biotech) according to the manufacturer’s instructions. The number of cells treated with different concentrations of MitoQ were counted under a microscope. Ethidium bromide (EtBr) pretreatment was utilized to reduce the mtDNA level of IEC-6 cells at indicated times, as previously described^10,23^.

### Measurement of intestinal permeability

Mice were fasted for 4 h and then received fluorescein isothiocyanate (FITC)-dextran (FD-40; 4 kDa; Sigma) by oral gavage (600 mg kg^−1^). Mice were sacrificed by cervical dislocation after 4 h and bled by cardiac puncture. Fluorometry was used to detect serum FITC levels. The in vitro permeability of the mouse intestine was measured by performing the chamber analyses as described previously^24^.

### Bacterial translocation

Using aseptic techniques, mesenteric lymph nodes (MLN) and caudal lymph nodes (two samples for each mouse) were taken. After collected tissue samples were weighed, 0.1 g of each node was homogenized in a tissue grinder with 0.9 ml of sterile saline. Homogenates were diluted, and 100 μl dilutions were cultured on Mac-Conkey’s agar (Sigma-Aldrich, St Louis, MO, USA) at 37 °C for 24 h. Additionally, blood (obtained by cardiac puncture) and PF were obtained for bacterial colony counts by diluting and plating on LB agar (Sigma-Aldrich, St Louis, MO, USA) at 37 °C for 24 h. Bacterial growth on the plates was expressed as colony-forming units/g of tissue. Culture results were considered positive when more than 10^2^ colonies/g of tissue were observed, as previously described^3^.

### Immunofluorescence

The location and expression of occludin, claudin-1, ZO-1, and Nrf2 proteins were evaluated using immunofluorescence. Intestinal tissues were removed and washed immediately, mounted in embedding medium, and stored at −80 °C until use. Frozen sections (10 μm) were cut and mounted on slides. Antibody dilutions (1:100) of rabbit polyclonal antibodies against occludin, claudin-1, ZO-1 (all from Abcam, Cambridge, UK), and Nrf2 (Santa Cruz Biotechnology, Dallas, TX, USA) were incubated according to the manufacturer’s instructions. The sections were subsequently probed with their respective FITC-conjugated secondary IgG antibodies. Slides incubated in the absence of primary antibodies were used as negative controls. A confocal scanning microscope was used to perform confocal image analysis (Leica Microsystems, Heidelberg GmbH, Mannheim, Germany).

### Biochemical assays

Serum and tissue tumor necrosis factor (TNF-α), IL-1β, and IL-6 in mice and cell supernatants were detected using an enzyme-linked immunosorbent assay (ELISA) kit (R&D System). Alanine transaminase (ALT), lactic dehydrogenase (LDH), and creatinine contents (Nanjing Jiancheng, China) were measured using commercial kits according to the manufacturer’s instructions.

### ATP content determination

The Adenosine-50-triphosphate ATP Determination Kit (Beyotime) was used to detect ATP concentration according to the manufacturer’s instruction. ATP concentration were calculated by construction of a standard ATP calibration curve.

### Apoptosis detection

Terminal deoxynucleotidyl transferase dUTP nick end labeling (TUNEL) was used to detect apoptosis in the intestine and IEC-6 cells according to the manufacturer’s instructions. The proportion of TUNEL-positive cells quantified the apoptotic index, as previously described^3^. The Annexin-V/propidium Iodide (PI) Apoptosis Detection kit (BD Biosciences, Franklin Lakes, NJ) was also used to detect apoptosis according to the manufacturer’s protocol, as previously described^10^.

### Assessment of oxidative stress and mitochondrial transmembrane potential

Oxidative stress in the intestinal mucosa and IEC-6 cells was measured using MDA, SOD, GSH, and GSH-Px (Nanjing Jiancheng, China) according to the manufacturer’s recommendations. The fluorescent probes DCFH-DA and MitoSOX Red (Sigma) were used to measure the accumulation of intracellular ROS or mitochondrial ROS. A JC-1 fluorescent probe was used to evaluate mitochondrial transmembrane potential in IEC-6 cells. Briefly, the cells were plated in 6-well plates and treated by H/R. After harvest, the cells were re-suspended in DCFH-DA, MitoSOX Red, or JC-1 solution at 37 °C for 30 min and detected by flow cytometry (Becton-Dickinson, USA).

### mtDNA copy number and transcription levels

Total DNA was extracted using the DNeasy Tissue Kit (Qiagen, Valencia, CA) to quantify mtDNA copy number. mtDNA was quantified by amplifying complex IV, and nuclear amplicons were generated by amplification of a GAPDH segment. mtDNA copy number was expressed as the mean mtDNA copy number relative to the nuclear genome. For the quantification of mtDNA mRNA, total RNA was extracted with Trizol reagent (Life Technologies Inc., Carlsbad, CA, USA), and the oligo (dT)-primed complementary DNA was used for reverse transcription of the purified RNA. NADH dehydrogenase subunits 1 (*ND1*) and cytochrome c oxidase subunit 3 (*COX3*) were used to reflected the transcript level of mtDNA. GAPDH was selected as the internal control. The primers for qPCR analyses of the relevant sequences are listed in Supplementary Table 1.

### Western blotting analysis

Protein samples exacted from tissue were separated by SDS-PAGE and transferred to PVDF membranes. The membranes were then incubated with antibodies against the protein of interest (occludin, claudin-1, ZO-1, Nrf2, HO-1, NQO-1, γ-GCLC, cleaved capase-3, cytochrome C, or TFAM) (Abcam) overnight at 4 °C. GAPDH, β-actin, or COX IV were used as internal controls. To analyze cytoplasmic cytochrome C content, cytoplasmic, and nuclear extracts were isolated from the cells for western blot analysis, as previously described. Notably, to detect mitochondrial TFAM levels, the Mitochondria Isolation Kit (Beyotime) was used to isolate mitochondria from intestines and IEC-6 cells according to the manufacturer’s instructions. COX IV was used as the loading control for mitochondrial fractions.

### RNA interference in IEC-6 cells

Double-stranded siRNA corresponding to the homologous sequence of the Nrf2 or HO-1 gene was used to reduce Nrf2 or HO-1 expression. Lipofectamine RNAiMAX reagent was used to conduct transfections according to the manufacturer’s recommendations. Transfected scrambled siRNA was used as the negative control. The mRNA expression of HO-1, NQO-1, and γ-GCLC were detected when IEC-6 cells were treated with Nrf2 siRNA by qPCR assay. The IEC-6 cells were transfected for 48 h prior to receiving various treatments for further analysis.

### Statistical analysis

Results are expressed as the means ± standard deviation (SD), and were analyzed by SPSS 17.0 (Chicago, IL, USA) and GraphPad Prism software 6.0 (La Jolla, CA, USA). Student’s *t*-test or one-way analysis of variance was performed to compare continuous variables between groups. Differences in survival rates between groups were analyzed using log-rank tests. All *P*-values < 0.05 are considered significant.

## Results

### MitoQ protects against intestinal I/R injury and improves survival

Upon examination of histological changes by HE staining, pretreatment with MitoQ preserved the integrity of morphological structures, and alleviated both neutrophil infiltration and hemorrhage in the I/R-injured intestine compared to the I/R group, illustrating the protective effect of MitoQ preconditioning on I/R-injured intestinal mucosal epithelial damage (Fig. 1b). Similarly, the histological scores of intestinal injury were markedly elevated following I/R injury, but were reduced by MitoQ (Fig. 1b).

The levels of serum I-FABP, ALT, creatinine, and LDH were used as markers of tissue injury to characterize the lesions induced by I/R^25^. Intestinal I/R significantly elevated the concentrations of I-FABP, ALT, AST, and LDH compared to the sham group. Pretreatment with MitoQ significantly reduced the levels of these tissue injury markers (Fig. 1c). In addition, MitoQ pretreatment significantly attenuated the increase in pro-inflammatory cytokines (TNF-α, IL-1β, IL-6) and neutrophil infiltration (MPO) induced by I/R (Supplementary Figure 1).

Since the above results demonstrated the protective effects of MitoQ, we performed another survival experiment. We compared survival over 72 h using the Kaplan–Meier method and the log-rank test. Six of 20 mice injected with MitoQ were dead 72 h post-intestinal I/R, while only 7 of 20 mice in the vehicle group were still alive 72 h after intestinal I/R. In general, the survival rate in the MitoQ+I/R group was significantly higher than that in the I/R group (Fig. 1d).

H/R-treated IEC-6 cells are generally used to mimic intestinal I/R in vivo. Pretreatment with MitoQ at various concentrations of 0.1, 0.5, and 1.0 μM significantly attenuated the decrease in IEC-6 cell viability caused by H/R in a dose-dependent manner (Fig. 1e), suggesting that MitoQ also protects enterocytes from I/R injury in vitro.

### MitoQ preserves intestinal permeability and prevents bacterial translocation during intestinal I/R

**Fig. 2:**
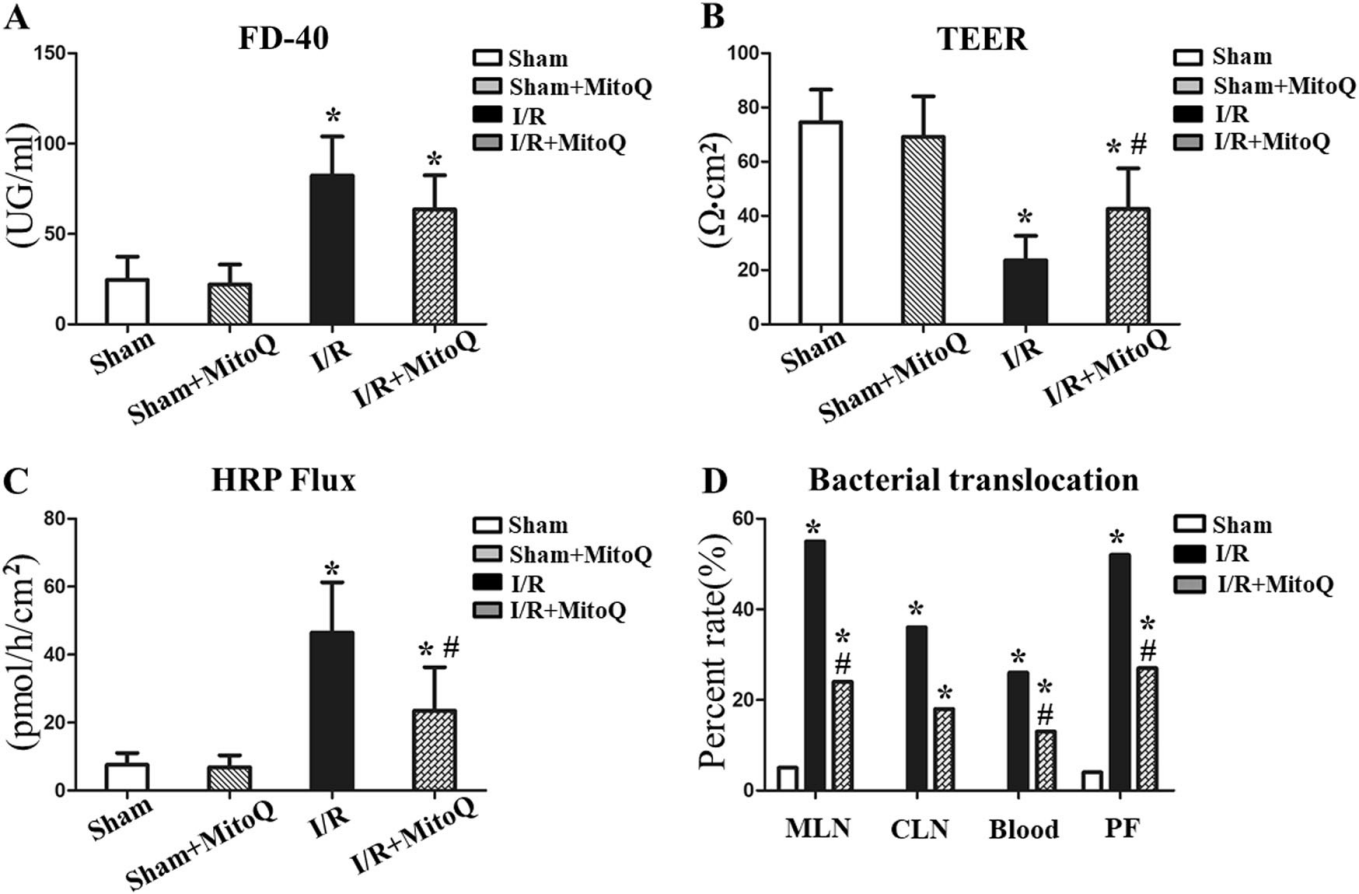
Effects of MitoQ on intestinal permeability and bacterial translocation. **a** Serum FD-40 was measured to evaluate in vivo permeability. **b**, **c** In vitro permeability of the mouse intestine measured by Ussing chamber analyses. MLN, CLN, blood, and PF were collected and cultured at 37 °C for 24 h. Culture results of bacterial growth were considered positive when more than 10^2^ colonies/g of tissue were observed. Data are expressed as the mean ± SD. ^*^P < 0.05 vs sham or control group; ^#^*P* < 0.05 vs I/R or H/R group

Our histological findings were paralleled by similar findings for intestinal permeability. Intestinal I/R resulted in increased permeability, as evidenced by higher levels of serum FD-40 (*P* < 0.01) and lower TEER (*P* < 0.01) compared to the sham group (Fig. 2a, b). The levels of FD-40 and intestinal permeability were both significantly reduced in the I/R+MitoQ group compared with those in the untreated I/R group. Accordingly, mice in the I/R group had decreased small bowel permeability resistance when compared with sham group, and resistance was increased in the MitoQ-treated group (Fig. 2c). These results indicate that MitoQ pretreatment decreases intestinal hyperpermeability following I/R injury.

Bacterial translocation to MLN, CLN blood, and peritoneal fluid (PF) serve as a readout for intestinal barrier integrity (Fig. 2d). Induction of intestinal I/R resulted in significant bacterial translocation to the MLN, CLN, blood, and PF. However, pretreatment with MitoQ dramatically decreased bacterial translocation to distant organs following I/R injury compared with untreated I/R mice.

### Effects of MitoQ pretreatment on intestinal tight junctions following I/R injury

**Fig. 3:**
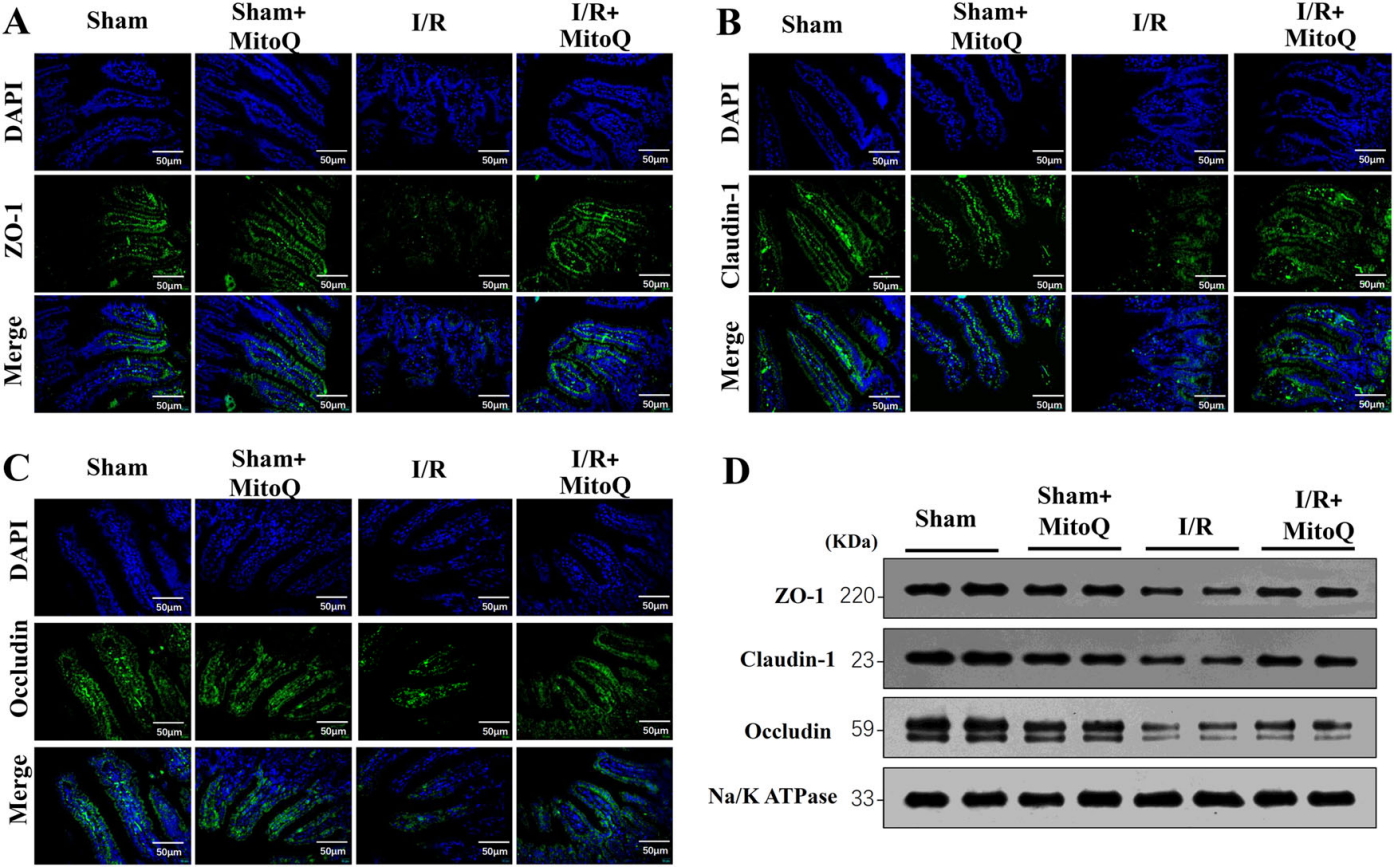
MitoQ restored the impaired intestinal barrier dysfunction in I/R-injured mice. Localization of ZO-1 (**a**), claudin-1 (**b**), occludin (**c**), and DAPI (DNA) within intestinal tissue sections assessed by immunofluorescence at 6 h after intestinal I/R. TJ proteins (green) DAPI stain (blue), and merged TJ proteins and DAPI images are presented. **d** The protein levels of occludin, claudin-1, and ZO-1 in intestinal mucosa were also measured by western blot at 6 h after I/R. *n* = 6 mice per group

As TJ proteins play a critical role in regulating intestinal epithelial permeability and maintaining barrier function^26^, redistribution of these protein leads to altered TJ structure; therefore, immunofluorescence and WB were used to evaluate TJ proteins of the intestinal mucosa. Biotin staining of TJ proteins (ZO-1, claudin-1, and occludin) showed a lack of focused staining in the lamina propria or deep within the surfaces of epithelial cells and some villi of the I/R-injured intestine, whereas MitoQ significantly attenuated these effects (Fig. 3a–c). Furthermore, WB analysis demonstrated that the concentration of TJ proteins was significantly decreased after I/R or H/R in vivo or in vitro, however, the expression of TJ proteins were increased in the I/R+MitoQ group (Fig. 3d). The proteins expression levels were quantified in Supplementary Figure 2.

### MitoQ reduces enterocyte apoptosis in response to intestinal I/R injury

**Fig. 4:**
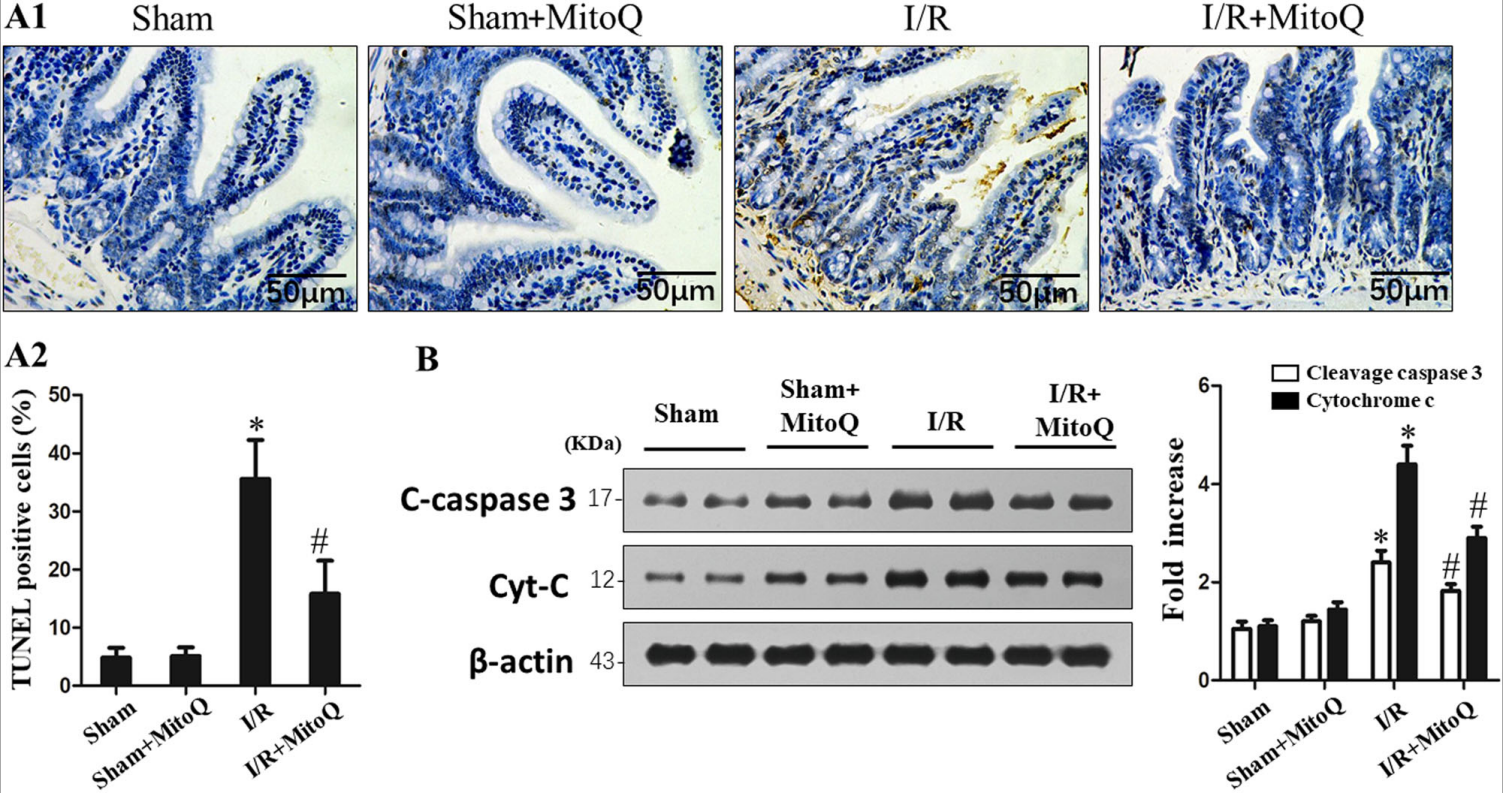
MitoQ attenuates intestinal I/R injury induced enterocyte apoptosis in vivo. **a**1 Representative images and **a**2 the apoptotic index of in situ TUNEL assay of intestinal epithelial cell apoptosis of mice. **b** Enterocyte apoptosis assessed by western blot of cleaved caspase-3 and cytochrome C. ^*^P < 0.05 vs sham group; ^#^*P* < 0.05 vs I/R. Values are expressed as the mean ± SD, *n* = 6

IEC apoptosis is thought to be one of the major accelerants of intestinal epithelial disruption^3^. To investigate the protective effects of MitoQ on enterocytes, TUNEL, cleaved caspase-3, and cytochrome C analyses were used to detect apoptosis in intestinal mucosa after intestinal I/R. Figure 4a shows that MitoQ pretreatment significantly reduced TUNEL-positive IECs. As shown in Fig. 4b, the cleaved caspase-3 and cytochrome C levels were significant in I/R-injured mucosa. Pretreatment with MitoQ significantly reduced cleaved caspased-3 and cytochrome C expression compared to I/R alone.

To further confirm the protective role of MitoQ in IEC apoptosis during I/R injury, TUNEL staining and flow cytometry analyses were used to evaluate IEC-6 cell apoptosis following H/R. TUNEL-positive apoptotic cells were significantly increased in the H/R group, while pretreatment with MitoQ reversed this effect (Supplementary Figure 3A). Annexin-V/PI analysis demonstrated that MitoQ alleviates H/R-associated cell death (Supplementary Figure 3B). In line with TUNEL and flow cytometry assays, the expression of cleaved caspase-3 and cytochrome C by western blot was decreased in the MitoQ-treated group compared with the H/R group (Supplementary Figure 3C).

### MitoQ protects against oxidative damage of mtDNA induced by I/R injury

**Fig. 5:**
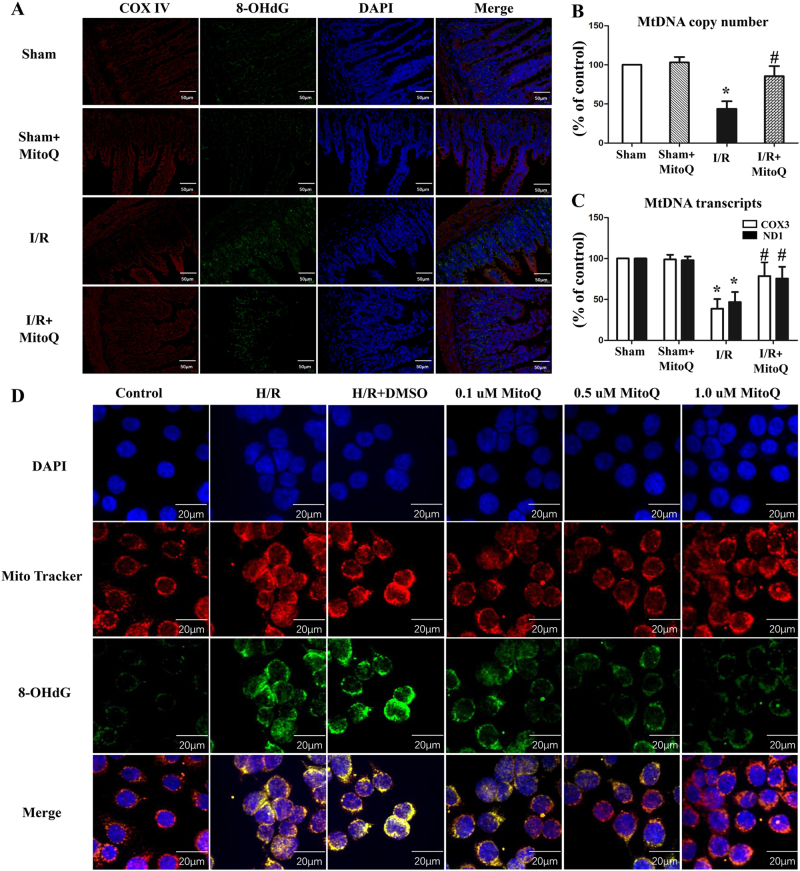
MitoQ attenuates mtDNA damage caused by intestinal I/R injury, both in vivo and in vitro. **a** Representative images of COX IV (green), 8-OHdG immunostaining (red), and DAPI (blue) in intestinal sections. The yellow in the merged images of green and red fluorescence indicates mitochondrial 8-OHdG-positive cells. **b**, **c** mtDNA copy number (**b**) and mtDNA transcript levels (**c**) in intestinal tissues analyzed by quantitative real-time PCR. mtDNA levels were normalized to the internal control GAPDH. Mitochondrial genes *ND*1 and *COX3* were chosen to indicate mtDNA transcription. **d** Representative images of MitoTracker red (red) and 8-OHdG immunostaining (green) in IEC-6 cells. ^*^P < 0.05 vs control; ^#^*P* < 0.05 vs I/R. Data are expressed as the mean ± SD. mtDNA mitochondrial DNA, COX IV cytochrome c oxidase subunit IV, 8-OHdG 8-hydroxyguanine, ND1 NADH dehydrogenase subunits 1, COX3 cytochrome c oxidase subunit 3

8-OHdG expression is reflective of DNA oxidative damage. Figure 5a shows that 8-OHdG-positive cells dramatically increased following I/R injury in the intestinal mucosa. Immunofluorescence analysis demonstrated co-localization of 8-OHdG and the mitochondrial protein COX IV in the cytoplasm of IECs, suggesting that intestinal I/R induces mtDNA point mutations in IECs. MitoQ preconditioning significantly ameliorated the I/R-induced oxidative damage of mtDNA in IECs. Furthermore, both mtDNA copy number and mtDNA transcript levels (*COX3* and *ND1*) were significantly reduced following intestinal I/R injury, effects which were prevented by MitoQ pretreatment (Fig. 5b, c).

Few 8-OHdG-positive IEC-6 cells were found in the control and MitoQ groups, while 8-OHdG-immunopositive cells significantly increased following H/R treatment (Fig. 5d). In addition, qPCR analyses also demonstrated that mtDNA copy number and mtDNA transcripts were reduced in the H/R group compared to the control (Supplementary Figure 4A). MitoQ pretreatment significantly prevented these changes.

We have demonstrated that oxidative damage of mtDNA leads to release of mtDNA into the cytoplasm, and even into the circulation. Circulating mtDNA, an indicator of the presence of damaged mitochondria^27^, significantly enlarges the inflammatory response during I/R injury. We, therefore, assessed the effects of MitoQ on circulating mtDNA levels following intestinal I/R injury. The results showed that I/R injury significantly elevated the circulating mtDNA levels and that MitoQ-pretreated mice exhibited markedly lower levels of circulating mtDNA compared with the I/R group (Supplementary Figure 4B).

To further explore the underlying mechanisms for mtDNA oxidative damage, we measure TFAM, a promoter-specific enhancer of mtDNA, a regulator of mitochondrial genes and mtDNA copy number, and a physical protector of mtDNA. The mitochondrial TFAM protein levels were dramatically reduced in the I/R-injured intestine compared with controls, which was prevented by MitoQ pretreatment. In addition, the expression of TFAM protein was markedly reduced in H/R-treated IEC-6 cells, which was also prevented by MitoQ preconditioning (Supplementary Figure 5A and 5B).

### Effects of MitoQ on oxidative stress and mitochondrial dysfunction during intestinal I/R injury

**Fig. 6:**
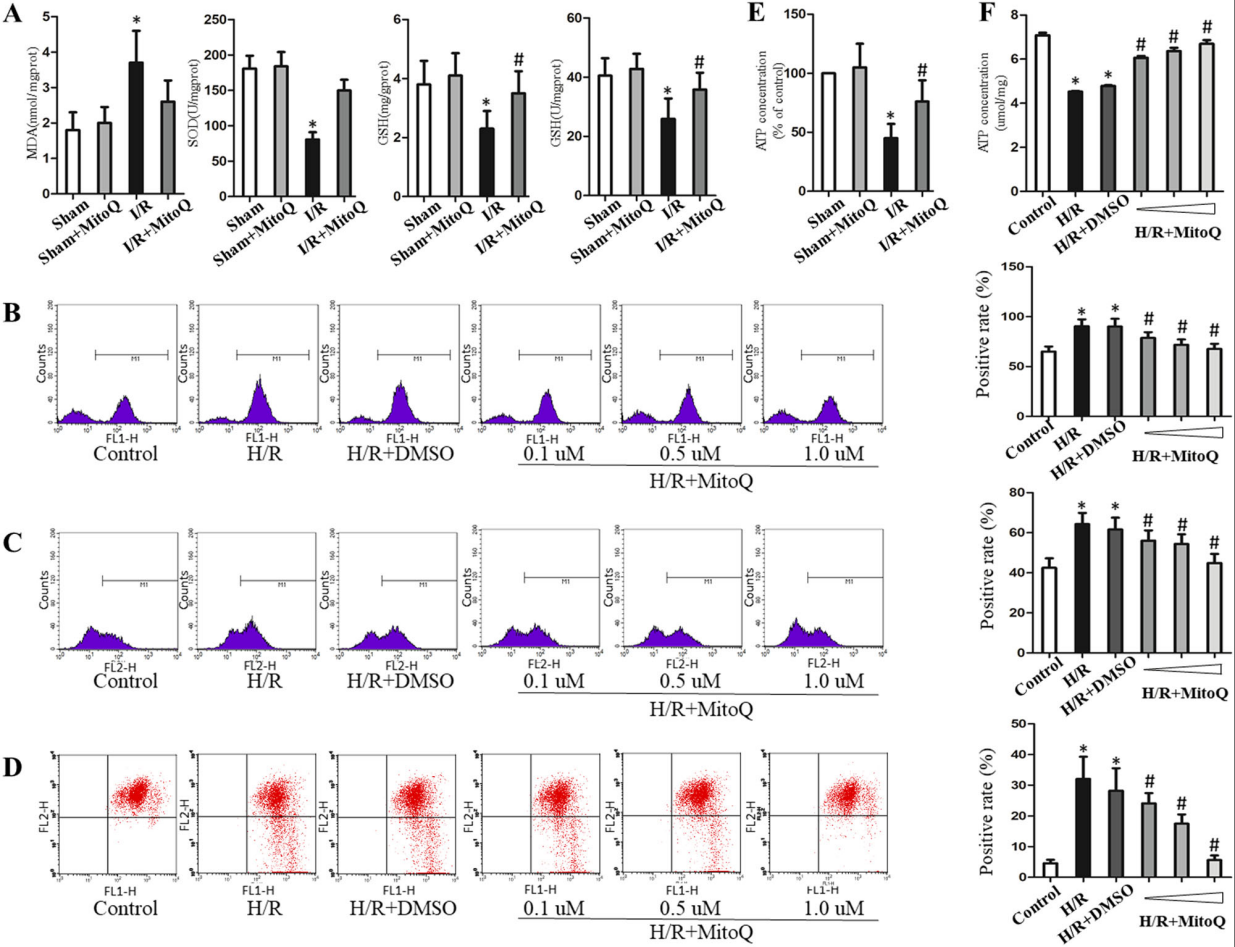
MitoQ alleviates oxidative stress and mitochondrial dysfunction in response to I/R injury. **a** MitoQ effects on MDA, SOD, GSH, and GSH-Px activities in intestinal tissue after I/R (*n* = 6). **b** Effects of MitoQ (0.1, 0.5, 1.0 μM) on cellular ROS levels in IEC-6 cells. **c** Effects of MitoQ (0.1, 0.5, 1.0 μM) on mitochondrial ROS levels in IEC-6 cells. **d** Effects of MitoQ (0.1, 0.5, 1.0 μM) on membrane potential (△Ψm) in IEC-6 cells. **e** ATP concentration quantified using a commercially available kit and expressed as a percentage of the sham group. **f** Effects of MitoQ on ATP content in H/R-treated cells. Data are expressed as the mean ± SD (*n* = 5). ^*^P < 0.05 vs sham or control group; ^#^*P* < 0.05 vs I/R or H/R group

Intestinal I/R injury increases oxidative stress. Figure 6a shows that MitoQ pretreatment markedly increased the levels of SOD, GSH, and GSH-Px and decreased the MDA level compared to the I/R group. To further confirm the protective role of MitoQ in oxidative stress and ROS production, H2-DCFDA and MitoSOX were assessed by flow cytometry analysis in IEC-6 cells following H/R. As shown in Fig. 6b, c, intercellular and mitochondrial ROS levels were both significantly increased in IEC-6 cells exposed to H/R, and MitoQ treatment significantly attenuated these effects.

We next quantified mitochondrial ATP, a biomarker of mitochondrial activity. Figure 6e indicates that ATP levels were markedly reduced following I/R injury, which was partially reversed by MitoQ preconditioning. To further evaluate the effects on mitochondrial function, the mitochondrial membrane potential (△Ψm) and cellular ATP levels were also measured in IEC-6 cells. After H/R treatment, flow cytometry analysis showed that △Ψm in IEC-6 cells was significantly decreased, and MitoQ pretreatment reversed this effect (Fig. 6d). MitoQ also partially reversed the decrease in ATP level caused by H/R (Fig. 6f).

### mtDNA depletion by EtBr induces mitochondrial injury and cell death

**Fig. 7:**
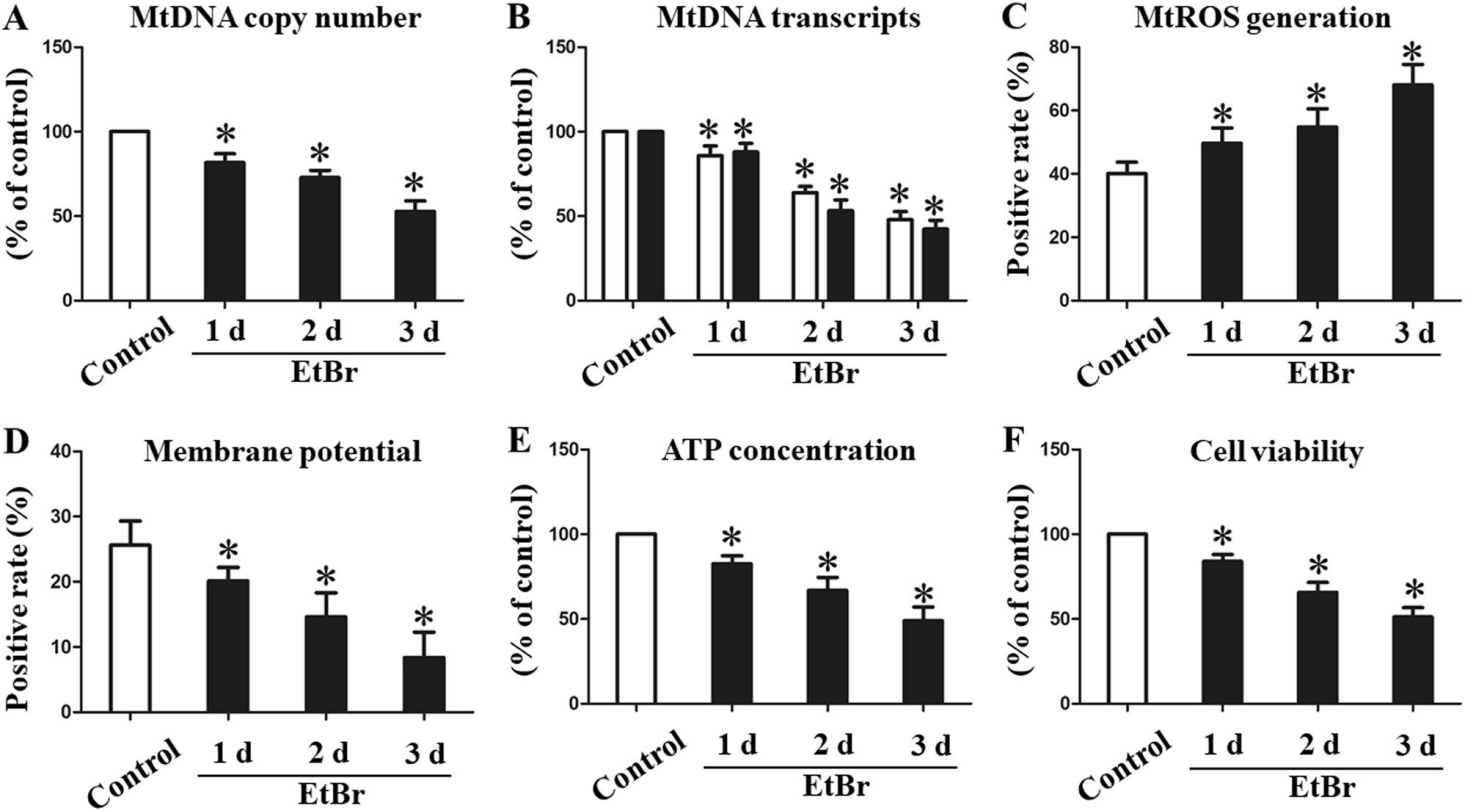
Depletion of mtDNA by EtBr-induced mitochondrial dysfunction and IEC-6 cell damage. **a** mtDNA copy number and **b** mtDNA transcript levels in IEC-6 cells quantified by real-time PCR after treatment with vehicle (control) or EtBr at the indicated times. Mitochondrial ROS (**c**),△Ψm (**d**), ATP content (**e**), and cell viability (**f**) quantification. ^*^P < 0.05 vs control. EtBr ethidium bromide

To investigate whether oxidative damage of mtDNA could induce mitochondrial disruption and cell injury, IEC-6 cells were exposed to low concentration EtBr to induce reduction of mtDNA content. Figure 7a, b show that the mtDNA copy number and mtDNA transcript levels were decreased by EtBr in a time-dependent manner, indicating mtDNA depletion. Mitochondrial ROS significantly increased in mtDNA-depleted cells, while ATP and △Ψm were significantly decreased (Fig. 7c–e). Furthermore, mtDNA depletion in IEC-6 cells time-dependently impaired the cell viability, suggesting that mtDNA depletion alone is sufficient to induce enterocyte death (Fig. 7f).

### MitoQ upregulates antioxidant genes via Nrf2/ARE activation

**Fig. 8:**
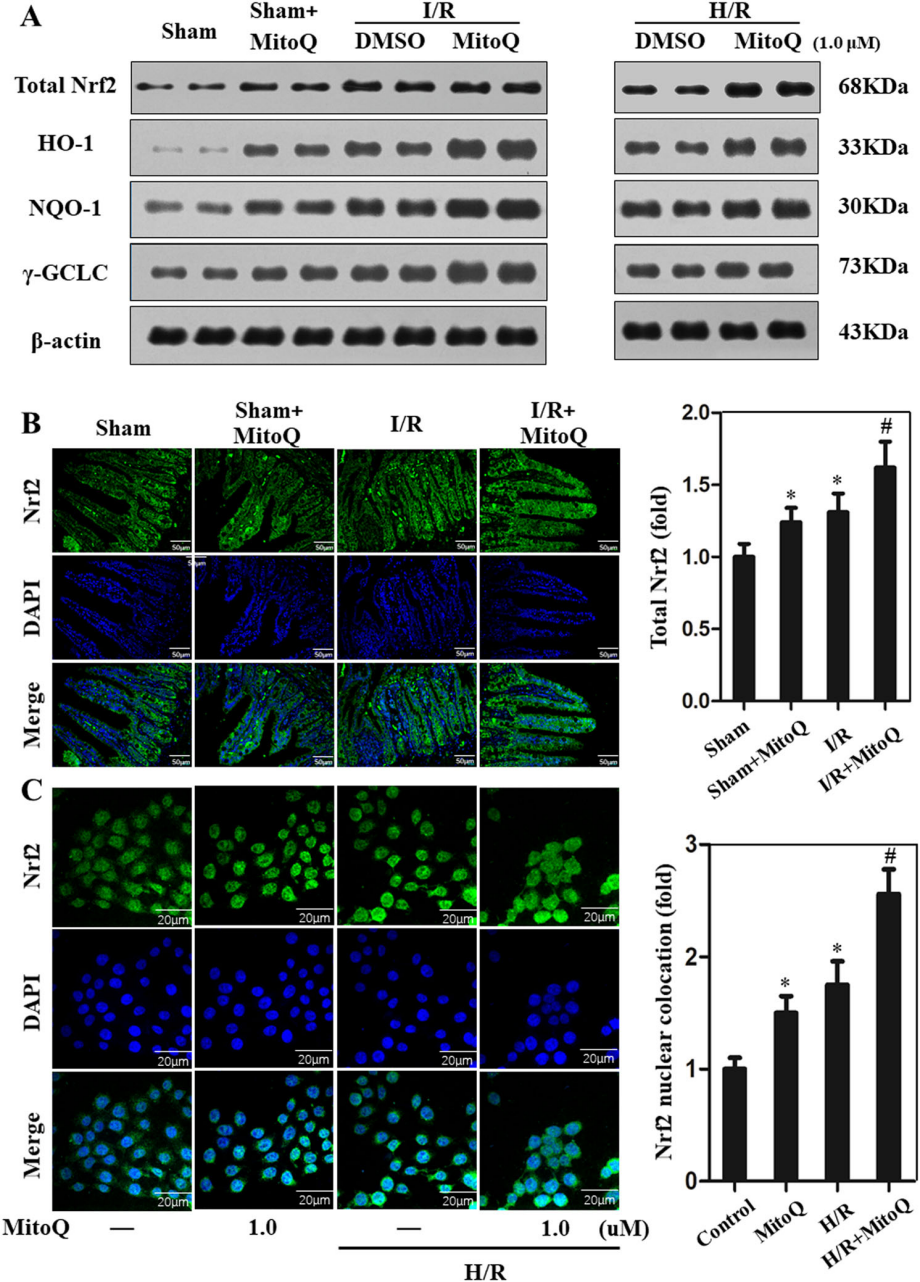
Effects of MitoQ on activation of the Nrf2 antioxidant pathway. **a** Western blot analysis of total Nrf2, HO-1, NQO-1, and γ-GCLC in MitoQ pretreatment mouse intestine 6 h after reperfusion and MitoQ-treated IEC-6 cells subjected to H/R injury. **b** Immunofluorescence analysis of the effect of MitoQ preconditioning on expression of Nrf2 after intestinal I/R injury. **c** Immunofluorescence staining showing changes in Nrf2 fluorescence. MitoQ increases nuclear translocation of Nrf2 (endogenous) in H/R-treated IEC-6 cells. ^*^P < 0.05 vs sham or control group; ^#^*P* < 0.05 vs I/R or H/R group

We hypothesized that the protective effects of MitoQ against oxidative stress and mitochondrial dysfunction could result from the activation of antioxidant genes following intestinal I/R injury. Western blot results demonstrated that MitoQ pretreatment of I/R-injured mice significantly increased Nrf2 levels compared with the I/R group in vivo and in vitro. Similar to the Nrf2 response, HO-1, NQO-1, and γ-GCLC were upregulated in mice with MitoQ pretreatment following intestinal I/R injury (Fig. 8a). The proteins expression levels were quantified in Supplementary Figure 6. To determine MitoQ-mediated nuclear translocation of Nrf2, nuclear import of Nrf2 into the intestinal mucosa and IEC-6 cells was monitored by immunofluorescence. As shown in Fig. 8b, c, nuclear Nrf2 was significantly increased in the MitoQ+I/R group compared with the sham and I/R+vehicle groups. Induction of antioxidant status by MitoQ depends on activation of Nrf2/ARE signaling. Therefore, we used the luciferase reporter assay to measure the promoter activity of ARE. The luciferase activity derived from the ARE promoter consistently increased after MitoQ treatment in a dose-dependent manner (data not shown). These data suggest the possible involvement of Nrf2 signaling in the protective effects of MitoQ against I/R-induced oxidative stress and suggest that MitoQ pretreatment promotes nuclear translocation of Nrf2 after intestinal I/R injury.

### MitoQ protects against mtDNA damage, oxidative stress, and apoptosis, in part via Nrf2 signaling

**Fig. 9:**
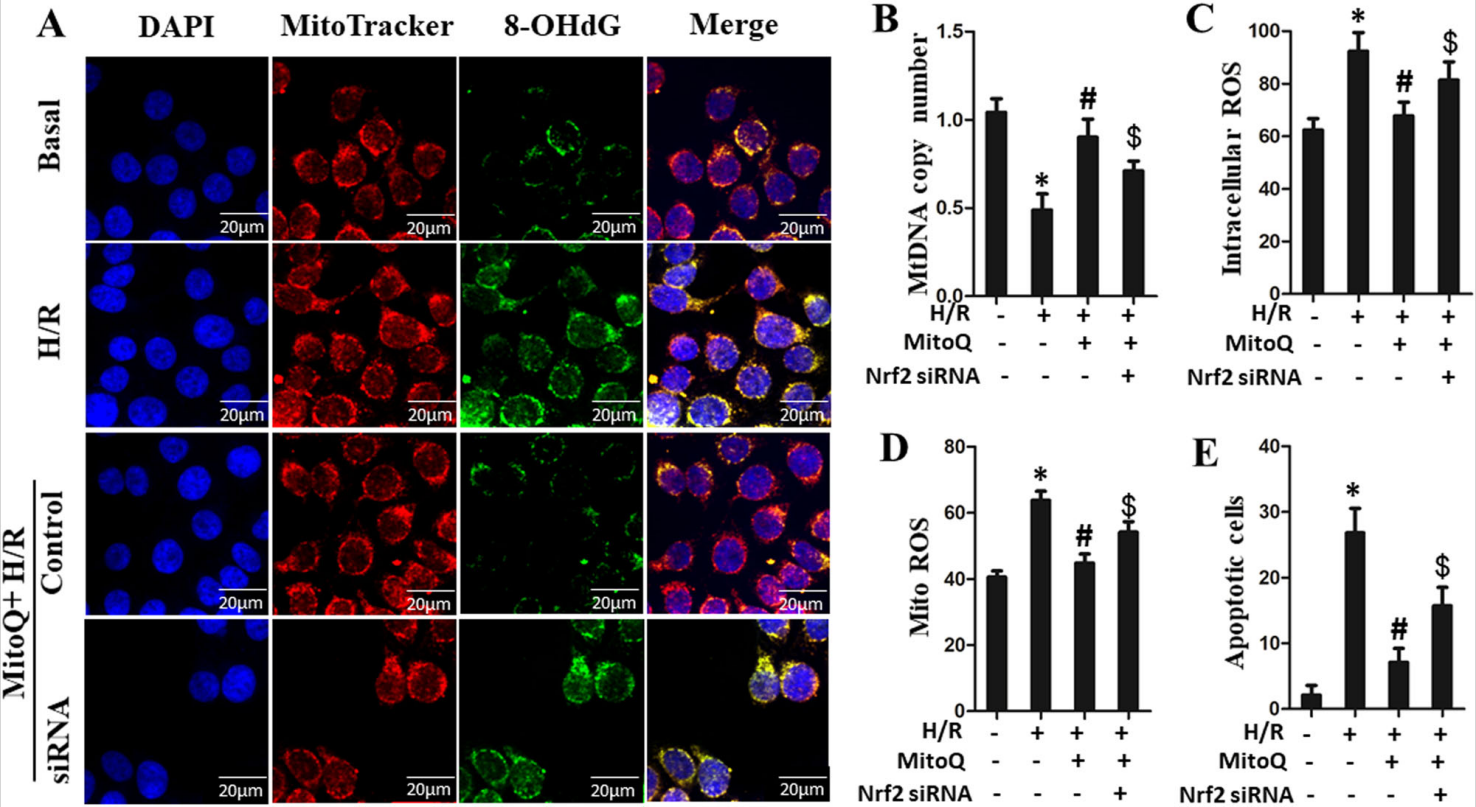
Protective effect of MitoQ on mtDNA damage, ROS production and apoptosis via Nrf2. IEC-6 cells were transfected with a specific siRNA against Nrf2 or a non-silencing control. **a** Confocal microscopic images showing that MitoQ ameliorates mtDNA oxidative damage in IEC-6 cells subjected to H/R treatment. This effect was partially blocked by Nrf2 siRNA. mtDNA copy number in IEC-6 cells analyzed by quantitative real-time PCR (**b**). **c**, **d**, **e**) Bar graphs represent intracellular ROS, mitochondrial ROS, and apoptosis levels, as demonstrated using DCFH-DA, MitoSOX, and TUNEL assays. ^*^P < 0.05 vs control group; ^#^*P* < 0.05 vs H/R group; ^$^*P* < 0.05 vs H/R+MitoQ group

We next blocked Nrf2 transcription by treating IEC-6 cells with Nrf2 siRNA and examined whether and how Nrf2 mediates the protective effects of MitoQ on enterocytes following H/R. As shown in Supplementary Figure 7A, Nrf2 siRNA resulted in efficient knockdown Nrf2 levels in IEC-6 cells. Furthermore, The mRNA levels of HO-1, NQO-1, and γ-GCLC were significantly decreased when IEC-6 cells were treated with Nrf2 siRNA by qPCR assay (Supplementary Figure 7A, 7B and 7C). Confocal imaging revealed that mtDNA oxidative damage was significantly increased in IEC-6 cells under H/R conditions, but was ameliorated by treatment with MitoQ. This effect was partially abolished by Nrf2 siRNA transfection (Fig. 9a, b). In accordance, MitoQ inhibited H/R-induced intercellular ROS, mitochondrial ROS, and apoptosis in IEC-6 cells. These effects were also attenuated by Nrf2 siRNA transfection (Fig. 9c–e). In addition, HO-1 siRNA was designed to directly test the protective actions of MitoQ. As shown in Supplementary Figure 8, HO-1 siRNA efficiently knocked down the HO-1 levels in IEC-6 cells and partially reduced the protective effects of MitoQ on mtDNA contents, oxidative stress, and apoptosis. These results suggest that MitoQ attenuates IEC-6 mtDNA damage, oxidative stress, and apoptosis in part via Nrf2 signaling.

## Discussion

In the present study, we demonstrated that MitoQ pretreatment could improves I/R-induced intestinal barrier injury. Phenotypically, MitoQ attenuated IEC necrosis and apoptosis, reduced oxidative stress, and ameliorated mitochondrial dysfunction in response to intestinal I/R injury. These effects were attributed to MitoQ-mediated protection against mtDNA oxidative damage. The protective effects of MitoQ on mtDNA may be attributed to mitochondrial ROS reduction and stabilization of mitochondrial TFAM. We also observed that the protective properties of MitoQ appeared to be accomplished in part by upregulation of cellular antioxidant genes, including HO-1,NQO-1, and γ-GCLC. These results indicate the existence of novel mechanisms, whereby MitoQ protects against I/R-induced intestinal barrier injury to maintain mitochondrial function and attenuate mtDNA oxidative damage partially via Nrf2/ARE signaling.

mtDNA is particularly susceptible to increased oxidative stress due to the lack of histone protection and limited capacity for DNA repair^9^. 8-OHdG levels prominently in response to mtDNA damage by excessive ROS. Accumulation of 8-OHdG not only induces more point mutations, but also decreases mtDNA replication and transcription. Additionally, depletions of mtDNA transcript levels and mtDNA copy number in IECs significantly impaired electron transport chain efficiency, hindered mitochondrial biogenesis, and disrupted mitochondrial function. In turn, mitochondrial dysfunction enhanced ATP depletion allowed an uncontrolled ROS surge and aggravated oxidative damage to mtDNA, including IEC apoptosis and necrosis that led to the disruption of TJ proteins and intestinal epithelial integrity (Supplementary Figure 9). The present study is the first to investigate the potential mechanism of mtDNA oxidative damage in mucosal barrier injury following intestinal I/R injury.

To our knowledge, there have been no studies focusing on the effect of MitoQ on intestinal barrier damage. The intestinal epithelial barrier is established by a single layer of intestinal epithelial cells and regulated by a number of intercellular junctional complexes. It is composed of apical cell membranes from enterocytes and intercellular TJ proteins, which help to prevent the invasion of harmful microorganisms, antigens, and toxins from the intestinal lumen into the lymphatic system and blood^28^. In the present study, we demonstrated that MitoQ attenuated intestinal barrier damage following I/R injury by alleviating mitochondrial dysfunction and mtDNA damage. Using intestinal permeability measurements, we showed that MitoQ protects against intestinal hyperpermeability in I/R-injured mice. Increased intestinal epithelial apoptosis in response to I/R injury was attenuated by MitoQ pretreatment and expression levels of TJ proteins, such as claudin-1, occludin, and ZO-1, were restored in mice that received MitoQ treatment. The inflammatory response has been suggested to disturb intestinal barrier integrity. We also demonstrated that MitoQ pretreatment downregulated pro-inflammatory cytokines in the intestinal mucosa following I/R injury.

In the present study, we discovered that the amount of 8-OHdG in the mtDNA results in more random point mutations and base mispairings. Accumulation of mtDNA point mutations beyond a certain threshold disrupts mitochondrial function^29^. In accordance with this statement, we found that I/R injury disrupted IEC mitochondrial integrity, decreased ATP formation, reduced △Ψm, and induced cytochrome c release. Furthermore, there was little increase of 8-OHdG content in the nuclear DNA of H/R-treated IECs, indicating that mtDNA is more vulnerable to I/R injury and oxidative stress than nuclear DNA. mtDNA copy number and transcription were dramatically reduced following intestinal I/R injury, and this depletion can be attributed to ROS-induced point mutations at the start of replication and transcription. Tan et al^30^. found that oxidative injury to mtDNA was detected after 1 h ischemia, whereas cell death was observed by TUNEL staining following 6 h of ischemia, suggesting that mtDNA oxidative damage may occur earlier than cell death and could lead to the initial injury. Therefore, inhibition of mtDNA damage could be a potent therapy in the early stages of the I/R injury process.

Although excessive ROS contribute to mtDNA damage, the mechanisms by which oxidative stress damages mtDNA during intestinal I/R injury remain unknown. TFAM is a nuclear-encoded protein that regulates transcription of the mitochondrial genome and binds mtDNA in a non-sequence specific fashion, facilitating its packaging into nucleoid structures. TFAM plays a critical role in maintaining mtDNA copy number and regulating mtDNA transcription and replication^10,31^. In addition, after reaching the mitochondrial matrix, TFAM promotes the formation of nucleoid structures to protect mtDNA from oxidative injury^32^. We showed that intestinal I/R reduced mitochondrial TFAM levels, which was prevented by MitoQ pretreatment.

MitoQ has been suggested to protect kidney and cardiac tissue after I/R injury due to its antioxidant function and inhibition of mitochondrial oxidative damage^13,14^. MitoQ is rapidly and extensively taken up by mitochondria, where it is converted to the active antioxidant ubiquinol form. In the present study, we demonstrated that MitoQ reduced intestinal I/R-induced mtDNA oxidative damage. Anna J et al^13^. demonstrated that MitoQ pretreatment attenuated oxidative stress and mitochondrial dysfunction following renal I/R injury. Therefore, MitoQ may decrease excessive ROS in I/R-injured IEC, and decrease oxidative damage to mtDNA. Additionally, MitoQ also reverses the reduction of mitochondrial TFAM expression that occurs in response to I/R injury. Either increased mitochondrial ROS or decreased TFAM expression alone can induce mtDNA oxidative injury, suggesting the possibility of cross talk between them. TFAM over-expression inhibits the production of ROS, while excessive ROS can reduce TFAM expression^10,31^. Thus, the protective effects of MitoQ in I/R injury may be due to both inhibition of mitochondrial ROS and stabilization of mitochondrial TFAM, which together contribute to the cyto-protection exerted by MitoQ.

In the present study, intestinal I/R resulted in a heightened systemic inflammatory response and neutrophil infiltration, as evidenced by the increased pro-inflammatory cytokines and MPO expression. Consistent with our findings, mitochondrial damage is known to contribute to the initiation of sterile inflammation and neutrophil accumulation during I/R injury^11^. One mechanism occurs through the release of mitochondrial components from damaged cells that act as mitochondrial damage-associated molecular patterns, such as oxidized mtDNA^33^. These molecules subsequently activate Toll-like receptor 9 and the inflammasome, leading to production of pro-inflammatory cytokines and neutrophil infiltration. MitoQ pretreatment of I/R mice reduced circulating IL-1β, IL-6, MPO expression, and mtDNA levels, suggesting that MitoQ alleviated the pro-inflammatory response by decreasing mitochondrial oxidative damage and subsequent mtDAMP release. This is important, because elevated circulating mtDAMP is suggested to exacerbate the inflammatory response, which can lead to inflammation-related secondary intestinal injury and remote organ injury^11^.

Activation of Nrf2/ARE signaling is a crucial factor in protecting cells from oxidative insults. Recent studies have suggested that coordinated upregulation of ARE-driven genes protects organs from I/R injury^34,35^. Accumulating evidence also suggests that the Nrf2/ARE pathway reduces apoptosis and alleviates the inflammatory response, resulting in a significant improvement in tissue retention and beneficial role in coordination^36^. A recent study suggested that MitoQ significantly reduces renal dysfunction after I/R injury through the Nrf2/ARE pathway^20^. To determine the mechanism by which pretreatment with MitoQ reduced intestinal I/R-induced oxidative stress, we investigated the effect of MitoQ on Nrf2 and antioxidant gene expression in vivo and in vitro. MitoQ pretreatment increased Nrf2 expression and promoted nuclear translocation of Nrf2 after intestinal I/R injury. In addition, upregulation of antioxidant genes (HO-1, NQO-1, and γ-GCLC) by MitoQ conferred cyto-protection against intestinal I/R-induced oxidative stress. We showed that Nrf2 siRNA partially reversed the protective effects of MitoQ, providing potent evidence for Nrf2/ARE as a cytoprotective signaling mechanism for MitoQ.

We also investigated the molecular mechanisms by which MitoQ modulated IEC mitochondrial function and mtDNA oxidative stress after intestinal I/R injury. Previous studies indicated that ROS levels and DNA damage significantly increase in Nfr2(−/−) mice, whereas elevation of Nrf2 leads to decreased ROS levels and DNA injury^37^. Kirtikar et al^38^. showed that fidarestat upregulates mitochondrial biogenesis and decreases mtDNA damage via the Nrf2/HO-1 pathway in colon cancer cells. Consistent with previous studies, we also found that Nrf2 inhibition partially reversed the protective effects of MitoQ in preventing damage of mtDNA and reduction of mtDNA transcription or mtDNA copy number, providing further evidence for Nrf2/ARE as a possible mechanism of mtDNA protection by MitoQ. Lipophilic cations, such as MitoQ are relatively lipid soluble, despite their net positive charge. As a result, they can pass easily and rapidly through phospholipid bilayers into mitochondria and do not require transport by ionophores or carrier proteins^39^. Considering that MitoQ can mediate cyto-protection in the cytoplasm and mitochondria, interference of Nrf2 only partially reversed the protective effects of MitoQ in IEC, providing a novel mechanisms for MitoQ protection against mtDNA damage via Nrf2/ARE. Despite our extensive efforts, the mechanism by which MitoQ modulates the Nrf2 signaling pathway remain to be delineated.

In conclusion, this study demonstrates the novel beneficial effects of MitoQ on mucosal barrier integrity after intestinal I/R injury using in vivo and in vitro models. The mechanisms underlying these effects may involve protecting IEC mtDNA from oxidative stress, a process associated with activation of Nrf2/ARE signaling, reduction of mitochondrial ROS and stabilization of mitochondrial TFAM, resulting in IEC injury and apoptosis attenuation after intestinal I/R injury. These findings strongly support the therapeutic value of MitoQ in intestinal barrier protection following I/R injury.

## Electronic supplementary material

Supplementary Figure 1(TIF 149 kb)

Supplementary Figure 2(TIF 108 kb)

Supplementary Figure 3(TIF 296 kb)

Supplementary Figure 4(TIF 180 kb)

Supplementary Figure 5(TIF 178 kb)

Supplementary Figure 6(TIF 194 kb)

Supplementary Figure 7(TIF 142 kb)

Supplementary Figure 8(TIF 133 kb)

Supplementary Figure 9(TIF 278 kb)

Supplementary Table 1(DOC 32 kb)

Supplementary Information

## References

[1] Matsuda A (2014). FK866, a visfatin inhibitor, protects against acute lung injury after intestinal ischemia-reperfusion in mice via NF-kappaB pathway. Ann. Surg..

[2] Liu Z (2016). MicroRNA-682-mediated downregulation of PTEN in intestinal epithelial cells ameliorates intestinal ischemia-reperfusion injury. Cell Death Dis..

[3] Wu X (2017). Systemic blockade of P2X7 receptor protects against sepsis-induced intestinal barrier disruption. Sci. Rep..

[4] Zhu Q, He G, Wang J, Wang Y, Chen W (2017). Pretreatment with the ALDH2 agonist Alda-1 reduces intestinal injury induced by ischaemia and reperfusion in mice. Clin. Sci..

[5] Peng Z, Ban K, Wawrose RA, Gover AG, Kozar RA (2015). Protection by enteral glutamine is mediated by intestinal epithelial cell peroxisome proliferator-activated receptor-gamma during intestinal ischemia/reperfusion. Shock.

[6] Gan X (2015). Propofol attenuates small intestinal ischemia reperfusion injury through inhibiting nadph oxidase mediated mast cell activation. Oxid. Med Cell Longev..

[7] West AP, Shadel GS (2017). Mitochondrial DNA in innate immune responses and inflammatory pathology. Nat. Rev. Immunol..

[8] Yu EP, Bennett MR (2016). The role of mitochondrial DNA damage in the development of atherosclerosis. Free Radic. Biol. Med.

[9] Mikhed Y, Daiber A, Steven S (2015). Mitochondrial oxidative stress, mitochondrial dna damage and their role in age-related vascular dysfunction. Int J. Mol. Sci..

[10] Yue R (2015). Mitochondrial DNA oxidative damage contributes to cardiomyocyte ischemia/reperfusion-injury in rats: cardioprotective role of lycopene. J. Cell Physiol..

[11] Hu Q, Wood CR, Cimen S, Venkatachalam AB, Alwayn IP (2015). Mitochondrial damage-associated molecular patterns (MTDs) are released during hepatic ischemia reperfusion and induce inflammatory responses. PloS ONE.

[12] Escribano-Lopez I (2016). The mitochondria-targeted antioxidant MitoQ modulates oxidative stress, inflammation and leukocyte-endothelium interactions in leukocytes isolated from type 2 diabetic patients. Redox Biol..

[13] Dare AJ (2015). Protection against renal ischemia-reperfusion injury in vivo by the mitochondria targeted antioxidant MitoQ. Redox Biol..

[14] Dare AJ (2015). The mitochondria-targeted anti-oxidant MitoQ decreases ischemia-reperfusion injury in a murine syngeneic heart transplant model. J. Heart Lung Transplant..

[15] Hu J. et al Nrf2 regulates the inflammatory response, including heme oxygenase-1 induction, by mycoplasma pneumoniae lipid-associated membrane proteins in THP-1 cells. *Pathogens and disease* 75(4) (2017). 10.1093/femspd/ftx044 28430965

[16] Xu D (2017). The triterpenoid CDDO-imidazolide ameliorates mouse liver ischemia-reperfusion injury through activating the Nrf2/HO-1 pathway enhanced autophagy. Cell Death Dis..

[17] Lau WL (2015). Role of Nrf2 dysfunction in uremia-associated intestinal inflammation and epithelial barrier disruption. Dig. Dis. Sci..

[18] Chen H (2014). Nrf2 deficiency impairs the barrier function of mouse oesophageal epithelium. Gut.

[19] Ferrari D (2016). Cyanidin-3-O-glucoside inhibits NF-kB signalling in intestinal epithelial cells exposed to TNF-alpha and exerts protective effects via Nrf2 pathway activation. Toxicol. Lett..

[20] Xiao L (2017). The mitochondria-targeted antioxidant MitoQ ameliorated tubular injury mediated by mitophagy in diabetic kidney disease via Nrf2/PINK1. Redox Biol..

[21] Miller DM, Singh IN, Wang JA, Hall ED (2015). Nrf2-ARE activator carnosic acid decreases mitochondrial dysfunction, oxidative damage and neuronal cytoskeletal degradation following traumatic brain injury in mice. Exp. Neurol..

[22] Chiu CJ, McArdle AH, Brown R, Scott HJ, Gurd FN (1970). Intestinal mucosal lesion in low-flow states. I. A morphological, hemodynamic, and metabolic reappraisal. Arch. Surg..

[23] Moro L (2009). Mitochondrial DNA depletion in prostate epithelial cells promotes anoikis resistance and invasion through activation of PI3K/Akt2. Cell Death Differ..

[24] Zuo L (2014). Cigarette smoking is associated with intestinal barrier dysfunction in the small intestine but not in the large intestine of mice. J. Crohns Colitis.

[25] Li Y (2017). 6-Gingerol protects intestinal barrier from ischemia/reperfusion-induced damage via inhibition of p38 MAPK to NF-kappaB signalling. Pharmacol. Res.

[26] Gonzalez Y, Aryal B, Chehab L, Rao VA (2014). Atg7- and Keap1-dependent autophagy protects breast cancer cell lines against mitoquinone-induced oxidative stress. Oncotarget.

[27] Hu Q (2017). Elevated levels of plasma mitochondrial DNA are associated with clinical outcome in intra-abdominal infections caused by severe trauma. Surg. Infect..

[28] Tong LC (2016). Propionate ameliorates dextran sodium sulfate-induced colitis by improving intestinal barrier function and reducing inflammation and oxidative stress. Front. Pharmacol..

[29] Yang JL, Weissman L, Bohr VA, Mattson MP (2008). Mitochondrial DNA damage and repair in neurodegenerative disorders. DNA Repair.

[30] Tan X (2013). Postconditioning ameliorates mitochondrial DNA damage and deletion after renal ischemic injury. Nephrol. Dial. Transplant..

[31] Stiles AR (2016). Mutations in TFAM, encoding mitochondrial transcription factor A, cause neonatal liver failure associated with mtDNA depletion. Mol. Genet Metab..

[32] Malarkey CS, Bestwick M, Kuhlwilm JE, Shadel GS, Churchill ME (2012). Transcriptional activation by mitochondrial transcription factor A involves preferential distortion of promoter DNA. Nucleic Acids Res.

[33] West AP, Shadel GS, Ghosh S (2011). Mitochondria in innate immune responses. Nat. Rev. Immunol..

[34] Ke B (2013). KEAP1-NRF2 complex in ischemia-induced hepatocellular damage of mouse liver transplants. J. Hepatol..

[35] Peake BF (2013). Hydrogen sulfide preconditions the db/db diabetic mouse heart against ischemia-reperfusion injury by activating Nrf2 signaling in an Erk-dependent manner. Am. J. Physiol. Heart Circ. Physiol..

[36] Slocum SL, Kensler TW (2011). Nrf2: control of sensitivity to carcinogens. Arch. Toxicol..

[37] Shi Z (2015). Reduction of DNA damage induced by titanium dioxide nanoparticles through Nrf2 in vitro and in vivo. J. Hazard Mater..

[38] Shukla K, Sonowal H, Saxena A, Ramana KV, Srivastava SK (2017). Aldose reductase inhibitor, fidarestat regulates mitochondrial biogenesis via Nrf2/HO-1/AMPK pathway in colon cancer cells. Cancer Lett..

[39] Lowes DA, Thottakam BM, Webster NR, Murphy MP, Galley HF (2008). The mitochondria-targeted antioxidant MitoQ protects against organ damage in a lipopolysaccharide-peptidoglycan model of sepsis. Free Radic. Biol. Med.

