# Supplementary figures and images for: The mitochondrially targeted antioxidant MitoQ protects the intestinal barrier by ameliorating mitochondrial DNA damage via the Nrf2/ARE signaling pathway
Source: Cell Death Dis. 2018 Mar 14;9(3):403. doi: 10.1038/s41419-018-0436-x (PMC5851994; doi:10.1038/s41419-018-0436-x)

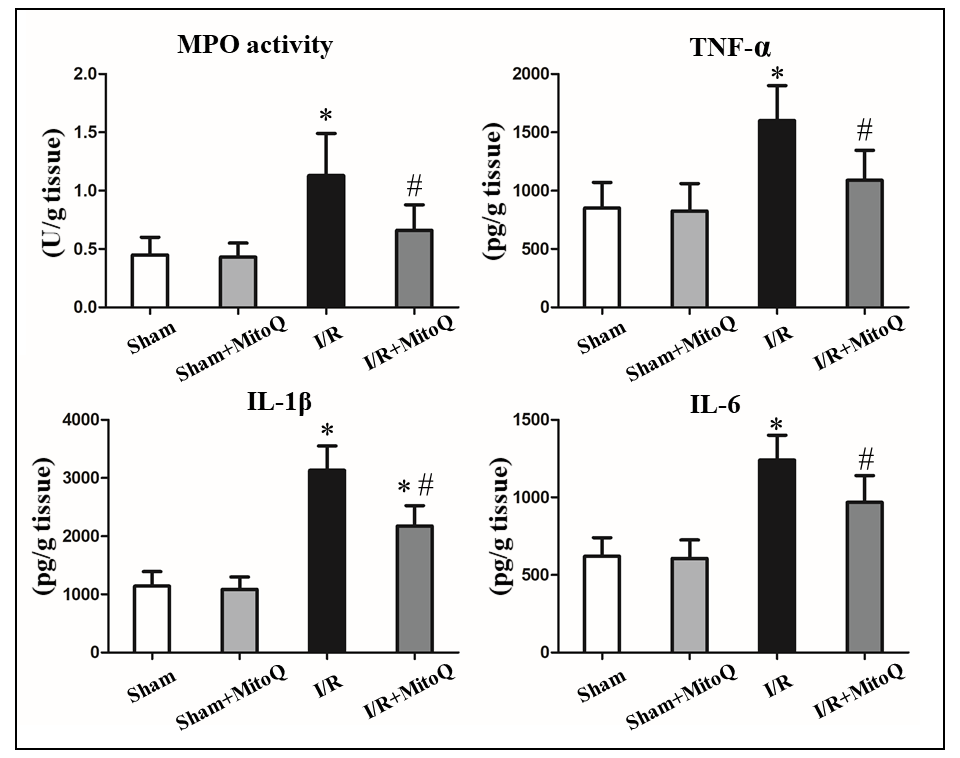

Supplement: Supplementary file 1 — Supplementary Figure 1(TIF 149 kb) [file 41419_2018_436_MOESM1_ESM.tif]

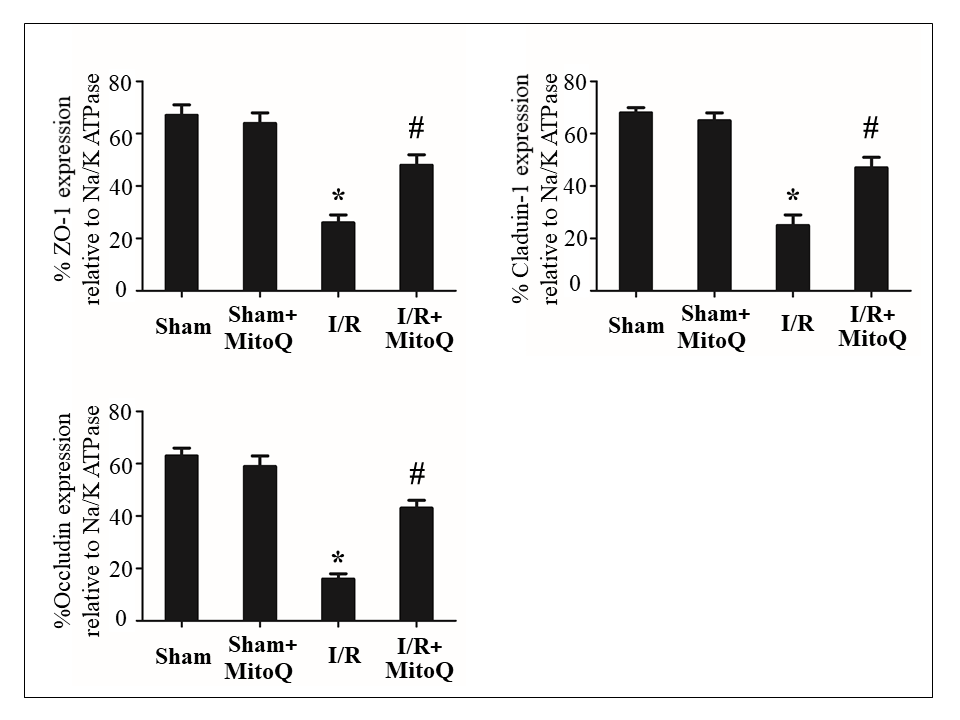

Supplement: Supplementary file 2 — Supplementary Figure 2(TIF 108 kb) [file 41419_2018_436_MOESM2_ESM.tif]

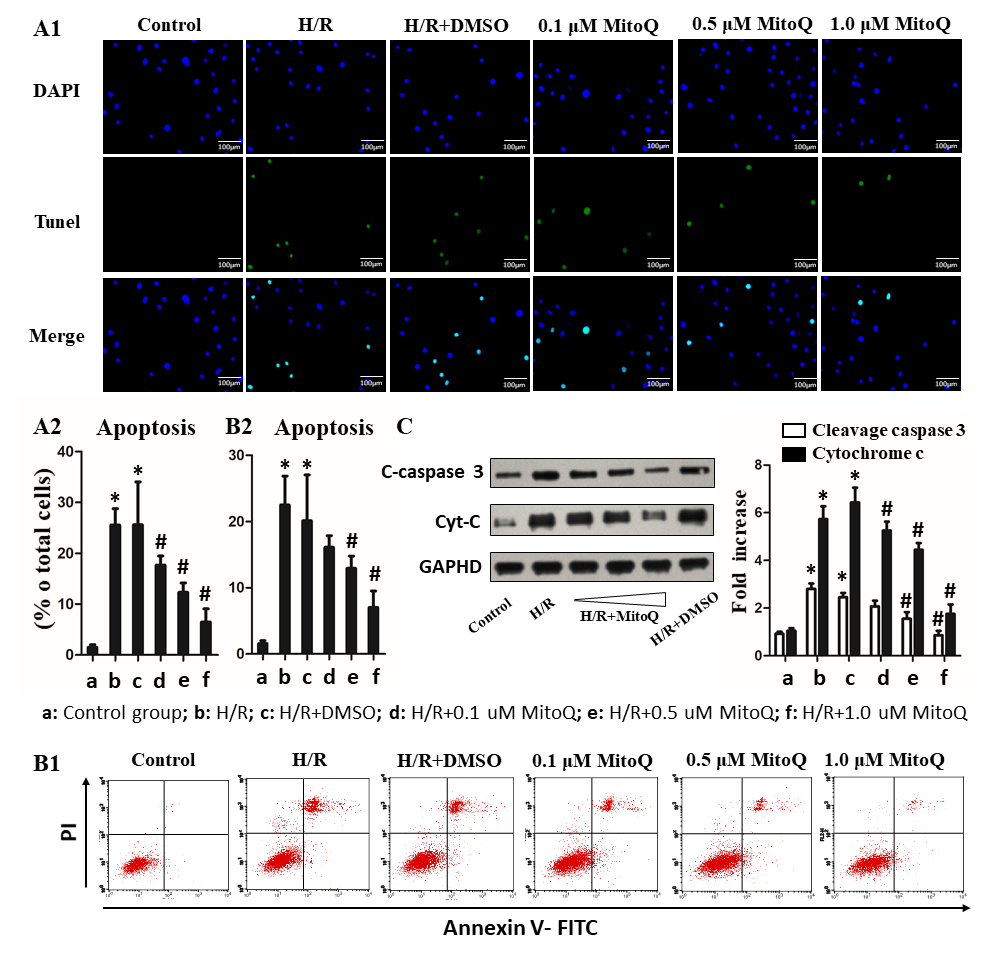

Supplement: Supplementary file 3 — Supplementary Figure 3(TIF 296 kb) [file 41419_2018_436_MOESM3_ESM.tif]

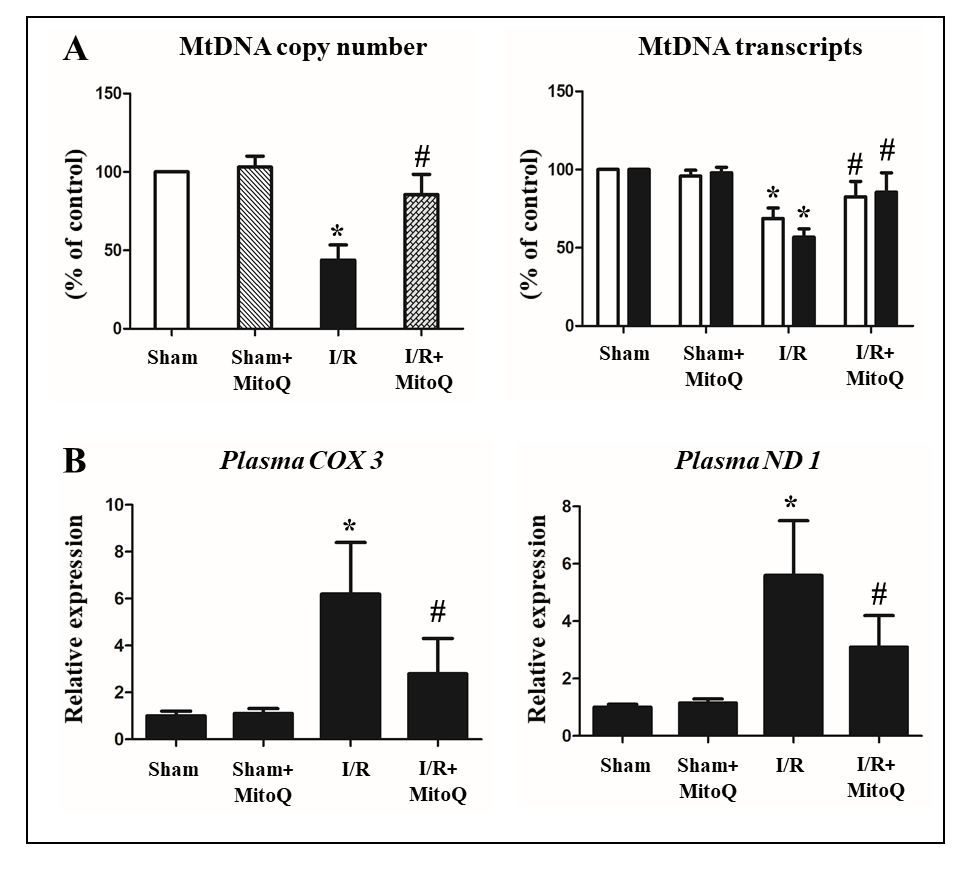

Supplement: Supplementary file 4 — Supplementary Figure 4(TIF 180 kb) [file 41419_2018_436_MOESM4_ESM.tif]

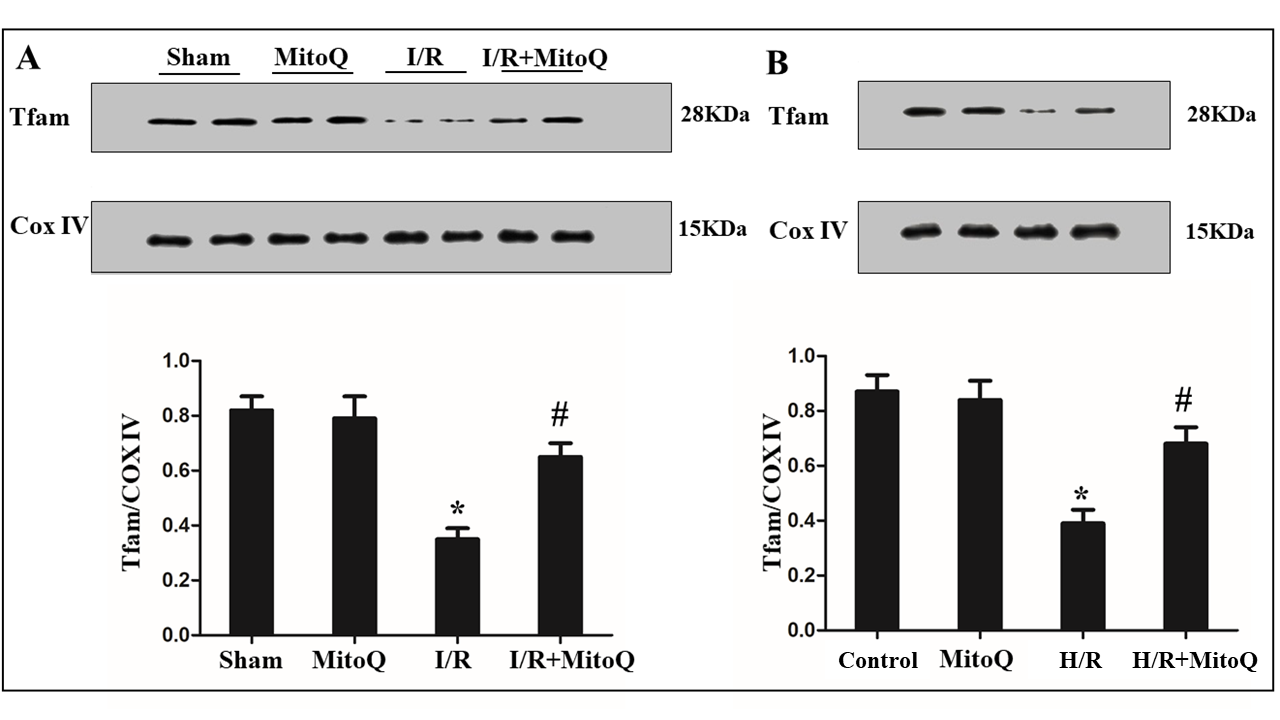

Supplement: Supplementary file 5 — Supplementary Figure 5(TIF 178 kb) [file 41419_2018_436_MOESM5_ESM.tif]

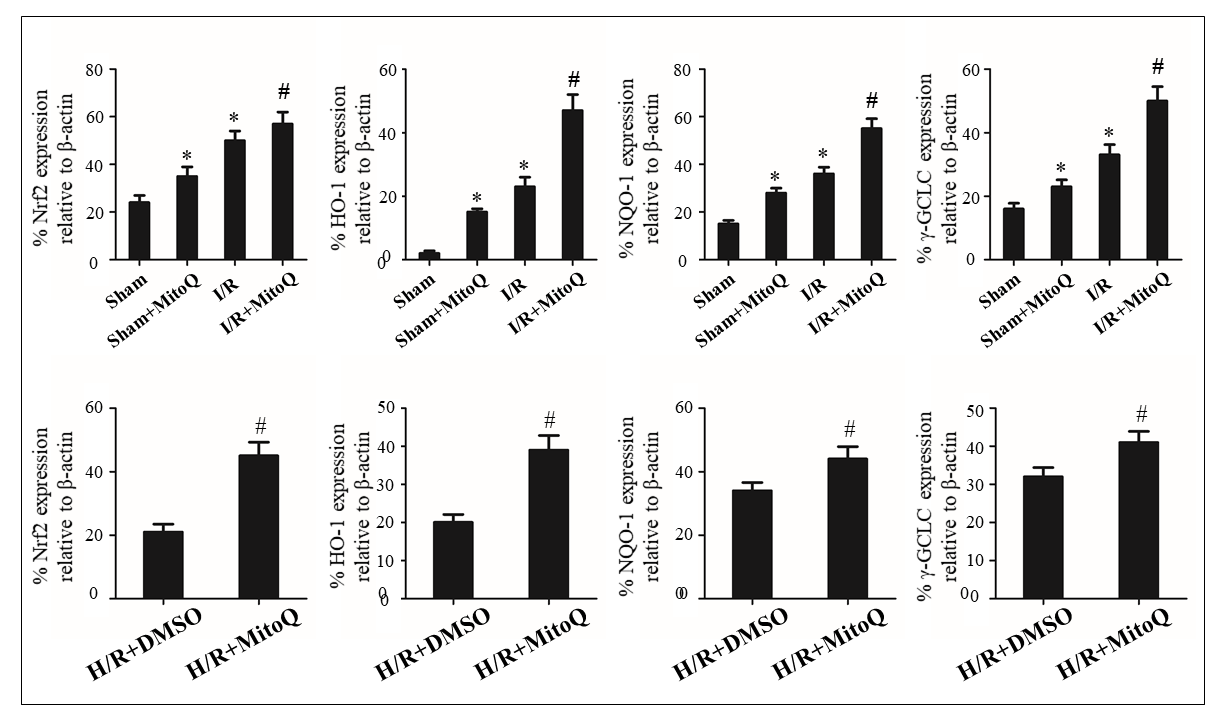

Supplement: Supplementary file 6 — Supplementary Figure 6(TIF 194 kb) [file 41419_2018_436_MOESM6_ESM.tif]

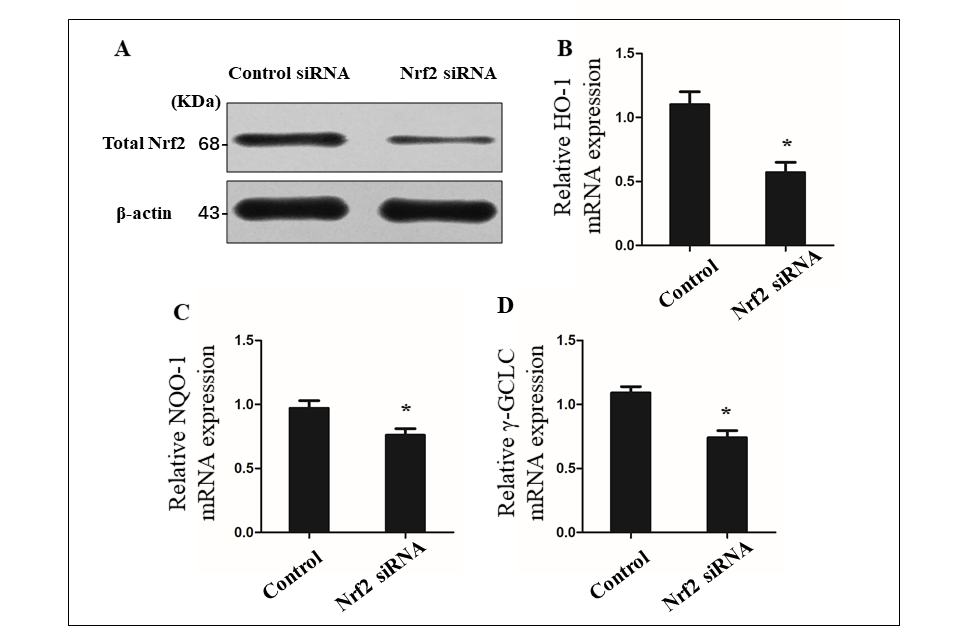

Supplement: Supplementary file 7 — Supplementary Figure 7(TIF 142 kb) [file 41419_2018_436_MOESM7_ESM.tif]

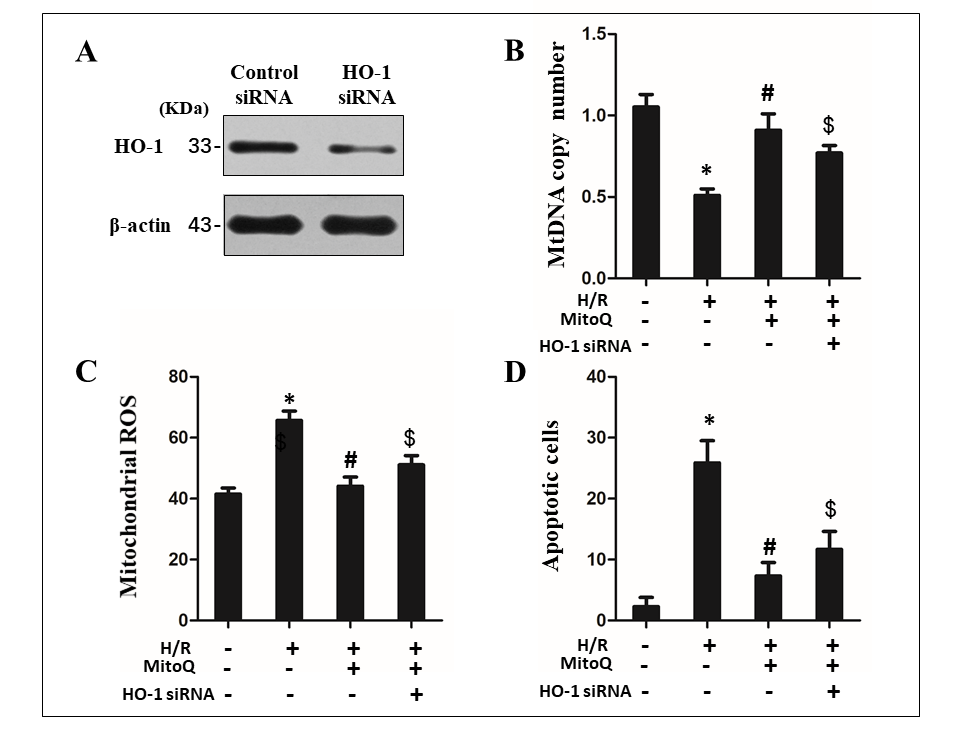

Supplement: Supplementary file 8 — Supplementary Figure 8(TIF 133 kb) [file 41419_2018_436_MOESM8_ESM.tif]

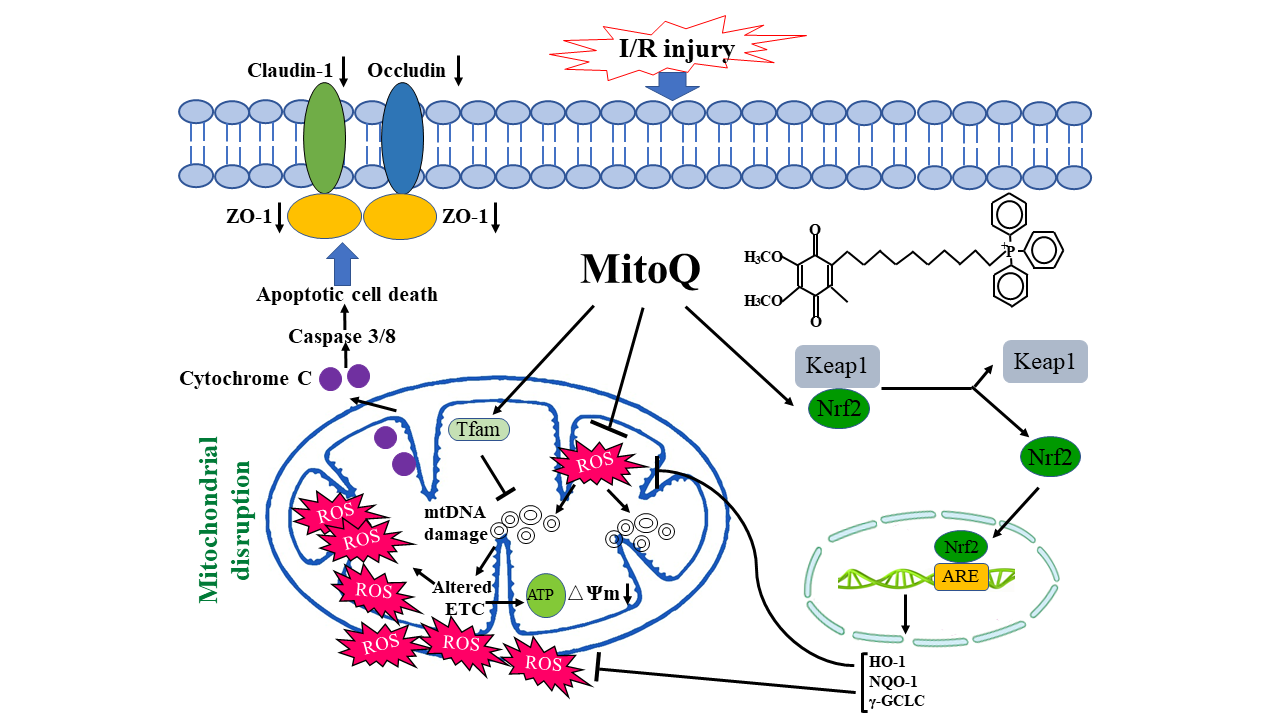

Supplement: Supplementary file 9 — Supplementary Figure 9(TIF 278 kb) [file 41419_2018_436_MOESM9_ESM.tif]
