# Supplementary material for: The mitochondrially targeted antioxidant MitoQ protects the intestinal barrier by ameliorating mitochondrial DNA damage via the Nrf2/ARE signaling pathway
Source: Cell Death Dis. 2018 Mar 14;9(3):403. doi: 10.1038/s41419-018-0436-x (PMC5851994; doi:10.1038/s41419-018-0436-x)
Supplement: Supplementary file 10 — Supplementary Table 1(DOC 32 kb) [file 41419_2018_436_MOESM10_ESM.doc]

Table S1. Primers used for quantitative PCR analysis

| **Genes** | **Primers sequence** |
| --- | --- |
| *Complex IV* | F: 5’-CCCCTGCTATAACCCAATACA-3’  R: 5’-CCAAACCCTGGAAGAATTAAGA-3’ |
| *Cox3* | F: 5’- CGTGAAGGAAACTACCCAGG -3’  R: 5’- CGCTCAGAAGAATCCTGCAA -3’ |
| *ND1* | F: 5’- GGATCCGAGCATCTTATCCA -3’  R: 5’- GGTGGTACTCCCTCTGTAAA -3’ |
| *HO-1* | F: 5’- CGTGCAGAGAATTCTGAGTTC -3’  R: 5’- AGACG CTTTACGTAGTGCTG -3’ |
| *NQO-1* | F: 5’- CGGTGAGAAGAGCCCTGAT -3’  R: 5’- CGACCACCTCCCATCCTT -3’ |
| *γ-GCLC* | F: 5’- TGAGATTTAAGCCCCCTCCT -3’  R: 5’- TTGGGATCAGTCCAGGAAAC -3 |
| *GAPDH* | F: 5’-TGTTGCTGTAGCCATATTCATTGT-3’  R: 5’-CCATTCTTCCACCTTTGATGCT-3’ |
