# Supplementary material for: The mitochondrially targeted antioxidant MitoQ protects the intestinal barrier by ameliorating mitochondrial DNA damage via the Nrf2/ARE signaling pathway
Source: Cell Death Dis. 2018 Mar 14;9(3):403. doi: 10.1038/s41419-018-0436-x (PMC5851994; doi:10.1038/s41419-018-0436-x)
Supplement: Supplementary file 11 — Supplementary Information [file 41419_2018_436_MOESM11_ESM.docx]

**Supplementary Information**

**Supplementary Figure 1**

Figure S1. Inflammatory responses of intestinal tissues from each group. ^*^P<0.05 vs sham group; ^#^P<0.05 vs I/R group.

**Supplementary Figure 2**

Figure S2. The proteins expression levels were quantified. ^*^P<0.05 vs sham group; ^#^P<0.05 vs I/R group.

**Supplementary Figure 3**

Figure S3. MitoQ protects against IEC-6 apoptosis following H/R. (A1) Representative images and (A2) apoptotic index of *in situ* TUNEL assay of IEC-6 cells. TUNEL-positive is green and DAPI is blue. (B) Flow cytometry analysis of annexin-V FITC and propidium iodide staining in IEC-6 cells. (B) Enterocyte apoptosis assessment by western blot of cleaved caspase-3 and cytochrome C. ^*^P<0.05 vs control; ^#^P<0.05 versus H/R. Values are expressed as the mean ± SD, n=6.

**Supplementary Figure 4**

Figure S4. Characterization of mtDNA copy number, mtDNA transcripts, and circulating mtDNA levels from each group. ^*^P<0.05 vs sham group; ^#^P<0.05 vs I/R group.

**Supplementary Figure 5**

Supplementary S5. Expression of TFAM protein in mitochondria analyzed by western blot. ^*^P<0.05 vs sham or control group; ^#^P<0.05 vs I/R or H/R group.

**Supplementary Figure 6**

Figure S6. The proteins expression levels were quantified. ^*^P<0.05 vs sham group; ^#^P<0.05 vs I/R or H/R group.

**Supplementary Figure 7**

Figure S7. Nrf2 siRNA resulted in efficient knockdown Nrf2 levels in IEC-6 cells (A). The mRNA expression of HO-1 (B), NQO-1 (C), and γ-GCLC (D) were significantly decreased when IEC-6 cells were treated with Nrf2 siRNA. ^*^P<0.05 vs control group.

**Supplementary Figure 8**

Figure S8. HO-1 siRNA efficiently knocked down the HO-1 levels in IEC-6 cells (A). (B, C and D) Bar graphs represent mtDNA copy number, mitochondrial ROS and apoptosis levels. ^*^P<0.05 vs control group; ^#^P<0.05 vs H/R group; ^＄^P<0.05 vs H/R+MitoQ group.

**Supplementary Figure 9**

Supplementary Figure 9. Flow chart.
